# Faujasite-Type Zeolite Obtained from Ecuadorian Clay as a Support of ZnTiO_3_/TiO_2_ NPs for Cyanide Removal in Aqueous Solutions

**DOI:** 10.3390/ijms24119281

**Published:** 2023-05-25

**Authors:** Ximena Jaramillo-Fierro, Hipatia Alvarado, Fernando Montesdeoca, Eduardo Valarezo

**Affiliations:** 1 Departamento de Química, Facultad de Ciencias Exactas y Naturales, Universidad Técnica Particular de Loja, San Cayetano Alto, Loja 1101608, Ecuador; bevalarezo@utpl.edu.ec; 2 Ingeniería Química, Facultad de Ciencias Exactas y Naturales, Universidad Técnica Particular de Loja, San Cayetano Alto, Loja 1101608, Ecuador; hcalvarado@utpl.edu.ec (H.A.); famontesdeoca2@utpl.edu.ec (F.M.)

**Keywords:** adsorption, photocatalysis, cyanide, clay, faujasite, ZnTiO_3_/TiO_2_, nanoparticles

## Abstract

In this study, zeolites prepared by the hydrothermal method from Ecuadorian clay were combined with the precursor clay and with the semiconductor ZnTiO_3_/TiO_2_ prepared by the sol-gel method to adsorb and photodegrade cyanide species from aqueous solutions. These compounds were characterized by X-ray powder diffraction, X-ray fluorescence, scanning electron microscopy, energy-dispersive X-rays, point of zero charge, and specific surface area. The adsorption characteristics of the compounds were measured using batch adsorption experiments as a function of pH, initial concentration, temperature, and contact time. The Langmuir isotherm model and the pseudo-second-order model fit the adsorption process better. The equilibrium state in the reaction systems at pH = 7 was reached around 130 and 60 min in the adsorption and photodegradation experiments, respectively. The maximum cyanide adsorption value (73.37 mg g^−1^) was obtained with the ZC compound (zeolite + clay), and the maximum cyanide photodegradation capacity (90.7%) under UV light was obtained with the TC compound (ZnTiO_3_/TiO_2_ + clay). Finally, the reuse of the compounds in five consecutive treatment cycles was determined. The results reflect that the compounds synthesized and adapted to the extruded form could potentially be used for the removal of cyanide from wastewater.

## 1. Introduction

Cyanide (CN^−^ or CN) is one of the toxic chemicals found in wastewater discharges from chemical synthesis (herbicides, fertilizers, fibers, nylon resins, and pesticides), electroplating, fabrication of auto parts, food industry, metal finishing, mining (extraction of metals such as gold and silver), pharmaceuticals, photography, and steel tempering [1]. It has been estimated that the release of cyanide from industries is more than 14 million kg/year in the world [2]. Cyanide can exist in four forms: total cyanide (CNT), weak acid dissociable cyanide (cyanide WAD or CN WAD), strong acid dissociable cyanide (cyanide SAD or CN SAD), and free cyanide (CNL) [3]. CNT includes cyanide WAD and cyanide SAD (strong complexes), such as cyanides of cobalt ((Co(CN)_6_)_4_^−^), gold ((Au(CN)_2_)^−^), and iron ((Fe(CN)_6_)_3_^−^, (Fe(CN)_6_)_4_^−^). Cyanide WAD comprises CNL and weak and moderately strong cyanide metal complexes such as cyanides of cadmium ((Cd(CN)_4_)_2_^−^), copper ((Cu(CN)_2_)^−^, (Cu(CN)_3_)_2_^−^, (Cu(CN)_4_)_3_^−^), nickel ((Ni(CN)4)_2_^−^), silver ((Ag(CN)_2_)^−^), and zinc ((Zn(CN))_4_)_3_^−^). CNL includes CN^−^ and hydrogen cyanide (HCN) [4].

Cyanide in concentrations that exceed environmental regulations constitutes a very dangerous compound for humans, aquatic organisms, and the environment in general [5]. CN exerts an inhibitory action on certain metabolic enzymes. The main compound affected is the enzyme cytochrome c oxidase. The inhibition of CN on this enzyme causes the blockade of the electron transport chain at the mitochondrial level, preventing the absorption of oxygen so that between 0.5 and 3.5 mg of cyanide per kilogram of body mass in humans can cause death [6].

The United States Environmental Protection Agency (USEPA) has proposed a regulation for drinking water and aquatic water with respect to CNT of 0.2 and 0.05 mg/L, respectively, and in terms of CNL for protection of aquatic life in freshwater establishes it at 0.022 mg/L and 0.0052 mg/L [7]. The European Union has set a lower level of 0.05 mg/L of cyanides in environmental water [8] and has allowed maximum concentration of 0.07 mg/L for mineral waters [9]. Ecuadorian regulations (agreement no. 97/A of 2017, Annex 1 of Book VI, Ministry of the Environment) establish 0.2 mg/L of CN as a quality criterion for water for human and domestic consumption that requires conventional treatment, and for the preservation of aquatic and wildlife life in cold fresh water, warm fresh water, and marine and of estuary 0.1 mg/L of CN. This same norm establishes limits for discharge to the public sewage system of 1 mg/L of CNT, for discharge to a body of fresh water 0.1 mg/L CNT, and for discharge to a body of marine water 0.2 mg/L of CNT.

Liquid emissions that contain cyanide above the permissible limits are treated by chemical methods (mainly crystallization, electrocoagulation, catalytic oxidation, ozonation processes), physical (adsorption, photocatalytic, precipitation, flotation) and biological (bioremediation). The most common chemical methods include alkaline chlorination, the INCO process (International Nickel Company’s, SO_2_/air), hydrogen peroxide, Caro’s acid method (H_2_SO_4_/H_2_O_2_), ozonation, reverse osmosis, activated carbon and resins [10]. However, the chemical processes to degrade cyanide demand chemicals and form secondary pollutants, these pollutants need additional treatment before their removal, this disadvantage is added to the high costs and incomplete decomposition. What creates the need for the development of more economical, effective and sustainable methods and technologies to this environmental problem [11]. An alternative are the processes called advanced oxidation technologies (AOTs) for the degradation of species resistant to traditional methods. Among the TAOs, heterogeneous photocatalysis has proven to be an efficient method for the degradation of aqueous and gaseous pollutants [12].

Heterogeneous photocatalysis involves the acceleration of the photoreaction in the presence of a semiconductor photocatalyst exposed to light radiation in the Visible and UV range. One of the major applications of heterogeneous catalysis is photocatalytic oxidation until complete mineralization of the pollutant, mainly to CO_2_, H_2_O, NO_3_^−^, PO_4_^3−^, and halide ions [13]. One of the most important semiconductor oxides is titanium dioxide (TiO_2_) due to its photocatalytic activity, low cost, low toxicity, chemical stability, insolubility, and resistance to photocorrosion [14]. The photocatalytic oxidation of cyanide is capable of transforming into products such as cyanate (OCN^−^), which is approximately 1000 times less toxic than CN^−^. After achieving this conversion, cyanate can be completely oxidized to carbon dioxide and nitrates as final products [15].

Titanium dioxide is an inexpensive and abundant mineral composed of titanium and oxygen. This particulate dust contains various crystalline phases, such as rutile (space group P42/mnm(136)) and anatase (space group I41/amd(141)) with a tetragonal structure and brookite (space group Pcab(61)) with a rhombohedral structure. The crystalline structure of each phase depends on the bonding manner of octahedral blocks of (TiO_6_) in the crystalline network [16]. Each phase has its own unique properties and applications. Titanium dioxide nanoparticles (NPs) are obtained using physio-chemical and green routes, among which are chemical synthesis in the vapor phase, hydrothermal, controlled precipitation, sol-gel, and polymeric precursor (Pechini) [17]. TiO_2_ is the most popular photocatalyst; however, it has low sensitivity to the visible light spectrum. Zinc oxide (ZnO) is also inexpensive and has high activity as a photocatalyst in the presence of UV radiation [18] and under solar irradiation [19]. This oxide has a bandgap energy similar to that of TiO_2_ (3.2 eV for TiO_2_ and 3.4 eV for ZnO) [18]. Zinc titanate, also known as zinc titanium oxide, is an inorganic compound that exists in three main forms: ZnTiO_3_ (ZnO-TiO_2_), Zn_2_TiO_4_ (2ZnO-TiO_2_), and Zn_2_Ti_3_O_8_ (2ZnO-3TiO_2_) [20]. ZnTiO_3_ has a perovskite crystal structure, which consists of a three-dimensional network of corner-sharing oxygen octahedra. The zinc and titanium ions occupy the center of these octahedra, resulting in the chemical formula ZnTiO_3_. For the synthesis of zinc titanates, ZnO and TiO_2_ can be used as zinc and titanium precursors, respectively, resulting in the titanates Zn_2_TiO_4_, Zn_2_Ti_3_O_8,_ and ZnTiO_3_, the latter being a rhombohedral structure, in most cases [21]. Several methods have been employed for the synthesis of ZnTiO_3_, including solid-state reactions and sol-gel processes. In the solid-state method, stoichiometric amounts of ZnO and TiO_2_ are mixed and heated at high temperatures. The sol-gel method involves the formation of a precursor solution, followed by gelation, drying, and calcination [20]. ZnTiO_3_ exhibits a wide bandgap, typically around 3–3.5 eV [22]. Due to the wide energy bandgap, this semiconductor has various current and potential uses in catalysts, microwave insulators, luminous materials, nonlinear optics, solar cells, and gas sensors [23].

The widespread use of TiO_2_ is restricted due to some disadvantages such as low adsorption capacity, high aggregation tendency and hard recovery [24]. To mitigate these disadvantages, some studies investigate the support of TiO_2_ nanoparticles in certain matrices. The support serves as an adsorbent to enrich contaminants and then accelerate the photocatalytic rate, and the scattering effect of the support can inhibit the growth of TiO_2_ crystallite sizes [25]. Some of the investigated supports are activated carbon, graphene, minerals, molecular sieve and glass [26]. Natural minerals such as kaolinite, diatomite, montmorillonite and zeolite have been used, among these, zeolite has some advantages such as high surface area, chemical and thermal stability, in addition, the high hydrophobicity of zeolite can promote the photocatalytic activity of TiO_2_-zeolite photocatalyst [27].

Zeolites are microporous aluminosilicates structured in three-dimensional crystalline networks, these minerals are composed of tetrahedrons formed by a cation and four oxygen atoms, that is, TO_4_, where the cation (T) is mainly silicon (Si) or aluminum (Al) [28]. Since the tetrahedrons are interconnected, their formula is TO_2_ since adjacent tetrahedrons share oxygen. Because aluminum has lower charges than silicon, the inclusion of aluminum is chemically offset by the inclusion of potassium (K), sodium (Na) and calcium (Ca) or less frequently by lithium (Li), magnesium (Mg), strontium (Sr) and (Ba). A total of 255 types of zeolites have been identified by their structure, of which more than 40 are natural [29]. Among these, faujasite, named in honor of the French geologist Barthélemy Faujas de Saint-Fond, is a series of three minerals: faujasite-Ca (FAU-Ca, (Ca, Na, Mg)_5_ (Si, Al)_12_ O_24_·15H_2_O), faujasite-Mg (FAU-Mg, (Mg, Na, K, Ca)_5_ (Si, Al)_12_ O_24_·15H_2_O), and faujasite-Na (FAU-Na, (Na, Ca, Mg)_5_ (Si, Al)_12_O_24_·15H_2_O) [30].

The soils of Ecuador are highly diverse and contain a wide variety of clays [31]. However, there are few investigations on the use of Ecuadorian clays to support photocatalysts. In addition, both oxides (TiO_2_ and ZnO) can enhance their own photocatalytic activity by coupling TiO_2_-ZnO [32]. This improvement is due to reduced recombination of electron holes. Furthermore, the increased migration of photogenerated carriers also enhances this activity [33]; however, to date, no cyanide removal studies using ZnTiO_3_/TiO_2_ have been reported. These facts have motivated us to carry out this study with the aim of synthesizing faujasite-type zeolite from Ecuadorian clay and using these minerals as support for ZnTiO_3_/TiO_2_ nanoparticles, which will be used in cyanide removal in aqueous solutions.

## 2. Results

### 2.1. Characterization 

#### 2.1.1. XRD Analysis

**Figure 1:**
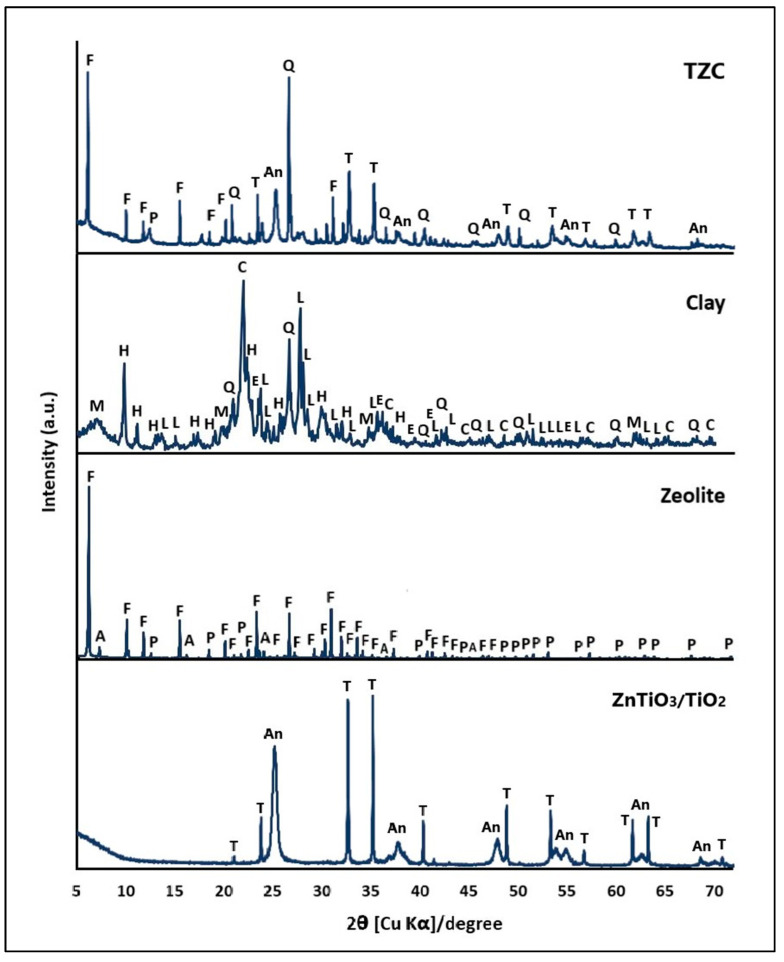
X-ray diffraction (XRD) pattern of TZC composite, clay, zeolite, and ZnTiO_3_/TiO_2_. M: montmorillonite, L: albite, C: cristobalite, Q: quartz, H: heulandite, E: hematite, F: FAU zeolite, A: LTA zeolite, P: Na-P1 zeolite, An: TiO_2_, and T: ZnTiO_3_.

Figure 1 presents the XRD patterns of the clay, the zeolite synthesized from this clay, and the ZnTiO_3_/TiO_2_ semiconductor. These materials were used as precursors to prepare extruded compounds.

Figure 1 shows that the mineral compound denoted as clay (C) is certainly composed of several phases, mainly by the phyllosilicate montmorillonite (M) and the tectosilicates albite (L), cristobalite (C), quartz (Q), hematite (E), and heulandite (H), while the zeolite consists mainly of FAU zeolite in sodium form with some impurities of LTA zeolites and Na-P1. The FAU zeolite was indexed to cubic phase with unit cell parameters a = b = c = 25.03 Å and space group Fd-3(203) according to the standard ICDD card no: 39-0218. The LTA zeolite was indexed to cubic phase with unit cell parameters a = b = c = 24.61 Å and space group Fm3c(226) according to the standard ICDD card no: 39-0222. The Na-P1 zeolite was indexed to the tetragonal phase with unit cell parameters a = b = 9.99 Å and c = 10.07 Å and space group I4_1_/amd(141) according to the standard ICDD card No: 44-0052. The zeolite sample presented a percentage of crystallinity of 85% compared to the standards, which was calculated according to Equation (14).

(1) $$\%\;\mathrm{Crystallinity}=\frac{\sum \mathrm{peak}\;\mathrm{areas}\;\mathrm{of}\;\mathrm{the}\;\mathrm{synthesized}\;\mathrm{samples}}{\sum \mathrm{standard}\;\mathrm{sample}\;\mathrm{peak}\;\mathrm{areas}}\times 100$$

Figure 1 also shows the main diffraction peaks of the hybrid semiconductor that consisted of 53% ZnTiO_3_ and 47% TiO_2_. The ZnTiO_3_ species was indexed to the hexagonal phase with unit cell parameters a = b = 5.08 Å and c =13.93 Å and space group R-3(148) according to the standard JCPDS card no. 00-015-0591 for the ZnTiO_3_ phase. On the other hand, the TiO_2_ (anatase phase) species was indexed to tetragonal phase with unit cell parameters a = b = 3.79 Å and c = 9.51 Å and space group I4_1_/amd (141) according to the standard JCPDS card no. 01-073-1764. When comparing the diffraction patterns of the extruded composites (after calcination) with their respective individual constituent (before calcination), no alteration of the respective diffraction patterns was observed. Therefore, the calcination temperature of 500 °C made it possible to achieve a mechanically strong adsorbent while keeping the crystalline structure of the zeolites and the photocatalyst intact.

The crystal size of the FAU and ZnTiO_3_/TiO_2_ zeolite particles was based on the main peak of the respective diffraction patterns. For the FAU zeolite, the peak (111) at position 2θ of 6.093 (d = 14.494 Å) was considered. The peak (104) at the 2θ position of 32.791 (d = 2.729 Å) and the peak (101) at the 2θ position of 25.302 (d = 3.517 Å) were considered for ZnTiO_3_ and TiO_2,_ respectively. The crystal size was calculated using the well-known Scherrer equation [34]:

(2) $$A=\frac{K\lambda }{\beta \;\cos\theta }$$

where K is the shape factor (here, K = 0.89), λ is the wavelength of the X-ray beam used (λ = 0.15405 nm), θ is the Bragg angle, and β is the full width at half maximum (FWHM) of the X-ray diffraction peak. The average crystal size of the FAU zeolite, ZnTiO_3,_ and TiO_2_ was 238.42 (±4.21) nm, 41.35 (±2.11), and 28.54 (±1.57) nm, respectively. The crystal size was also calculated using a Williamson–Hall plot. By plotting sinθ on the x-axis against βcosθ on the y-axis, it is possible to obtain the strain component of the slope and the particle size component of the intercept [35]. The crystal sizes estimated by this method were very close to those calculated with the Scherrer equation, thus for the FAU zeolite, ZnTiO_3_ and TiO_2_ the estimated values were 239.85, 42.36, and 29.29 nm, respectively. These variations in values are consistent with those reported by other authors [36,37]. The Williamson–Hall method was also used to estimate the lattice strain of the FAU zeolite, ZnTiO_3,_ and TiO_2_, obtaining values of 0.00045, 0.0013, and 0.0015, respectively.

Regarding the XRF analysis, Table 1 shows the main oxides present in the clay and zeolite and the composites. In Table 1, the XRF analysis showed the majority presence of the cations Al, Si, Fe, K, and Ca, in all the compounds, in addition to Ti and Zn, which were mainly incorporated as a mixed oxide of ZnTiO_3_/TiO_2_ in extrudates.

#### 2.1.2. SEM-EDX Analysis

**Figure 2:**
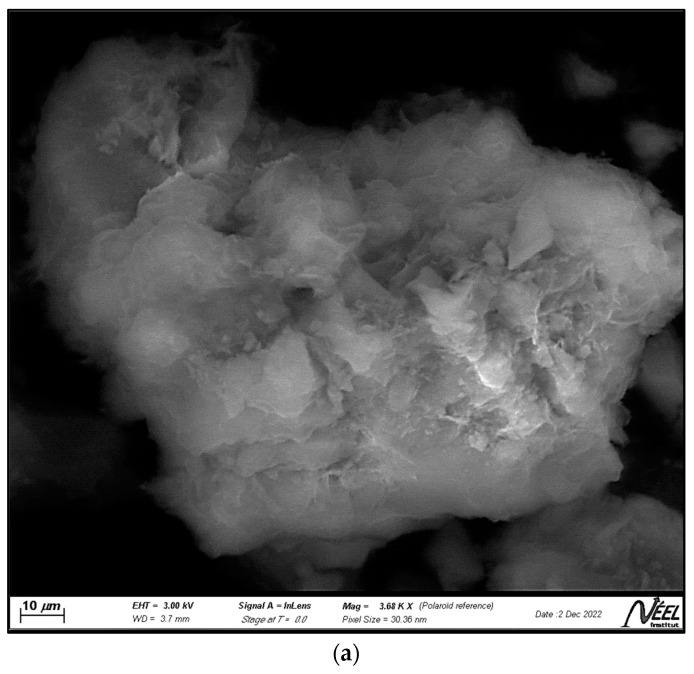

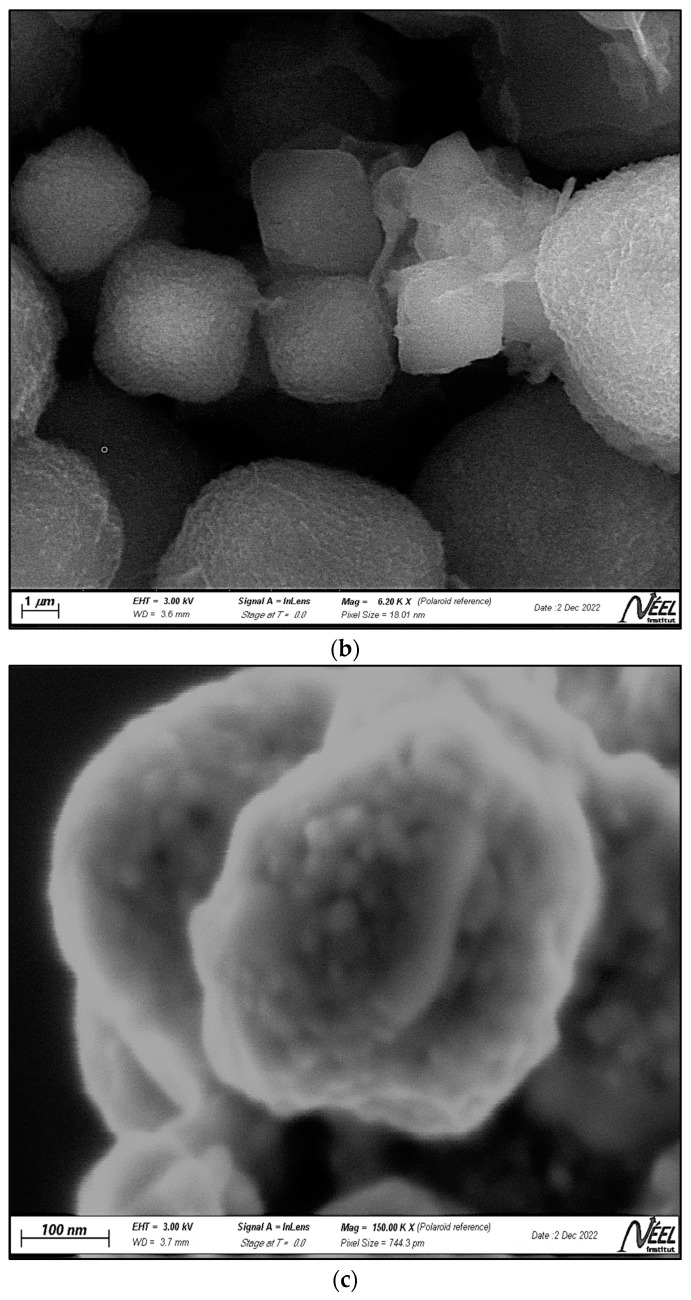

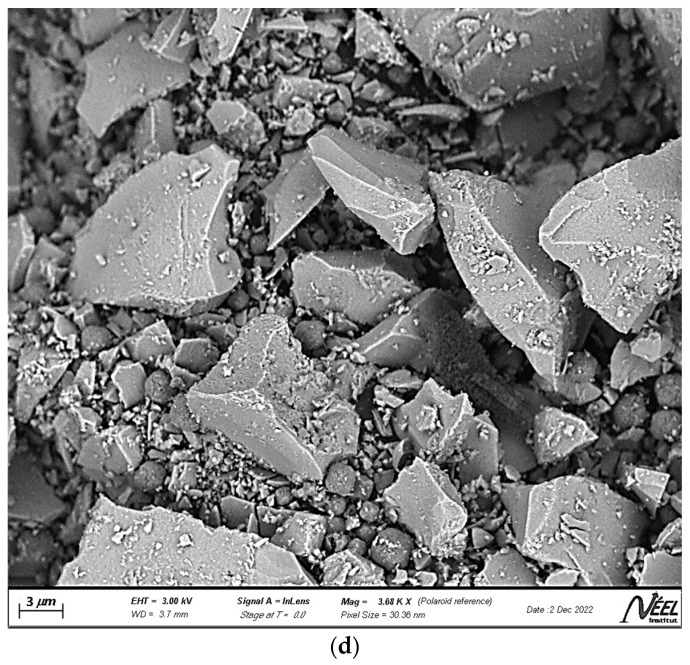
Scanning electron microscopy (SEM) images of (**a**) clay, (**b**) zeolite, (**c**) ZnTiO_3_/TiO_2_, and (**d**) TZC composite.

Figure 2a–c show the scanning electron microscopy (SEM) photomicrographs of the clay, the zeolite, and the ZnTiO_3_/TiO_2_ semiconductor, respectively. Figure 2a shows a clay surface formed by foliar and granular aggregates typical of the mineralogical phases that make up the clay, mainly montmorillonite, and albite. Figure 2b shows uniformly distributed cubic spheroidal granules, which correspond to FAU zeolite, while Figure 2c shows highly agglomerated, almost spherical ZnTiO_3_/TiO_2_ nanoparticles. The average particle size estimated from Figure 2b,c for the zeolite and ZnTiO_3_/TiO_2_ particles was 242.4 nm and 25.3 nm, respectively. 

**Figure 3:**
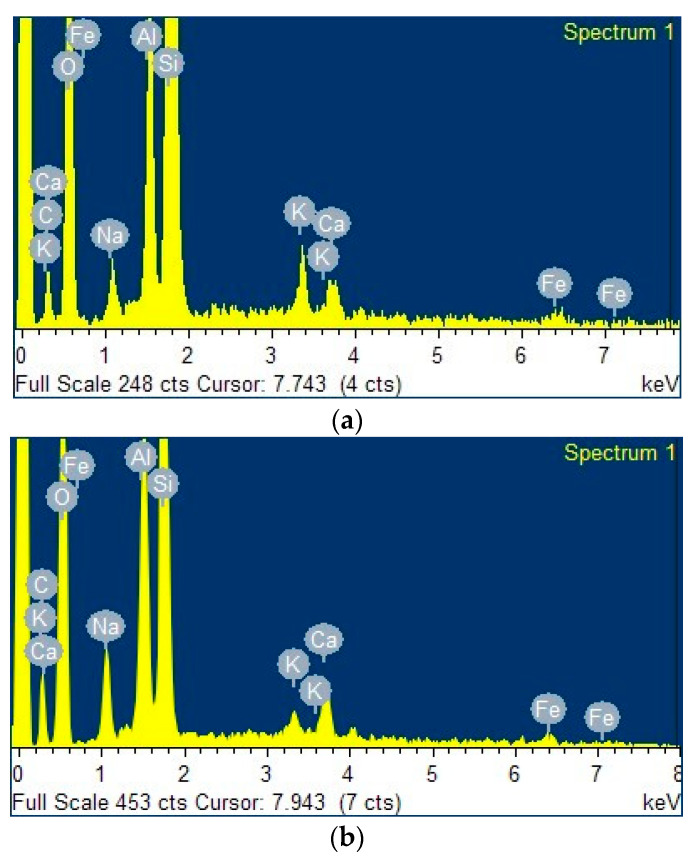

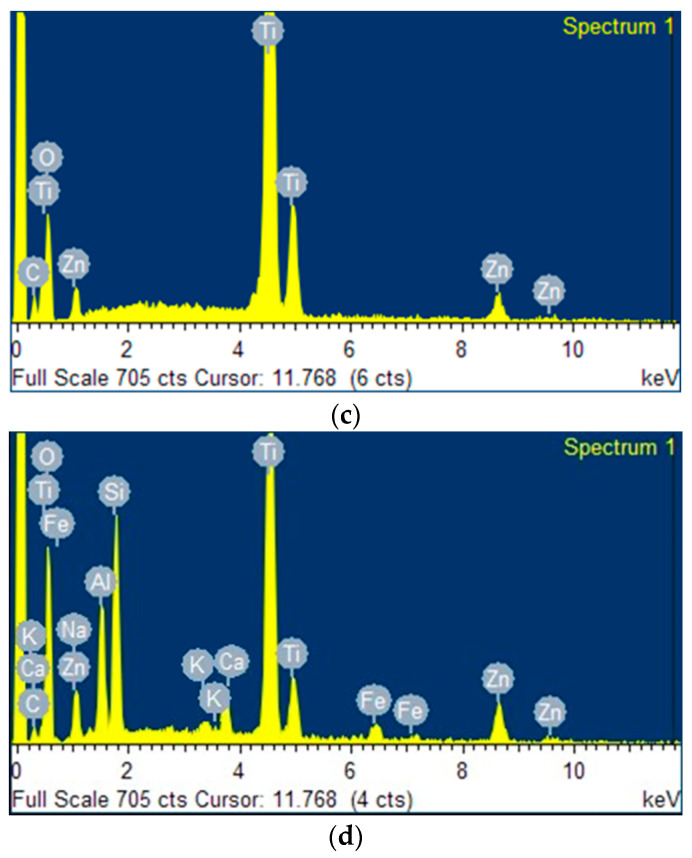
Energy-dispersive X-ray (EDX) spectra of (**a**) clay, (**b**) zeolite, (**c**) ZnTiO_3_/TiO_2_, and (**d**) TZC composite.

Figure 3a–c present the energy-dispersive X-ray (EDX) spectra of the clay, the zeolite obtained from this clay, and the ZnTiO_3_/TiO_2_ semiconductor, respectively. Both clay and zeolite have significant exchange cations, including Fe, Ca, Na, and K. Zeolite had a higher amount of sodium than their respective clay due to the calcination treatment carried out before synthesis. 

Table 2 shows the elemental composition (%) analyzed by EDX for the clay, the zeolite obtained from this clay, and the ZnTiO_3_/TiO_2_ semiconductor. From Table 2 it can be inferred that the Si/Al ratio of the synthesized zeolite was 2.13, which is in the range reported for the Y-FAU type sodium zeolite composition [38].

#### 2.1.3. Specific Surface Area (SSA) and Point of Zero Charge (PZC) Analysis

The specific surface area (m^2^ g^−1^) and the point of zero charge (pH_PZC_) of the samples in both powder (P) and extrudate (E) form are summarized in Table 3. As can be seen in this table, the clay has relatively low area values, probably because the crystalline phases present are dense tectosilicates (low porosity), such as albite, quartz, and cristobalite. Samples adapted to the extrudate form had a smaller specific surface area compared to samples in powder form due to the heat treatment required for their preparation. On the other hand, Table 3 also shows that the zeolite-based samples presented higher pH_PZC_ values than the other samples due to the incorporation of NaOH in the clay zeolitization process.

### 2.2. Adsorption Studies

**Figure 4:**
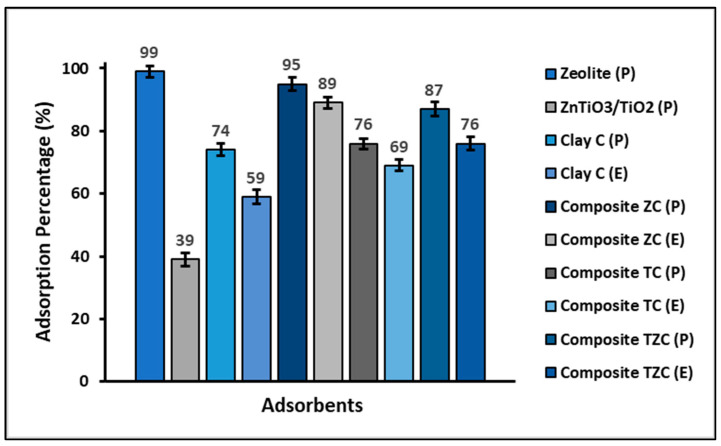
Percentage of adsorption of cyanide species on the powdered and extruded compounds. (Adsorbent = 200 mg L^−1^, KCN = 20 mg L^−1^, pH = 7.0 ± 0.1).

Figure 4 shows that clay in powder form, as well as ZC, TC, and TZC composites also in powder form, have better adsorption capacity than their extruded counterparts. In addition, the zeolite-based materials showed a higher adsorption capacity for cyanide species than the materials prepared without zeolite.

#### 2.2.1. Effect of pH

**Figure 5:**
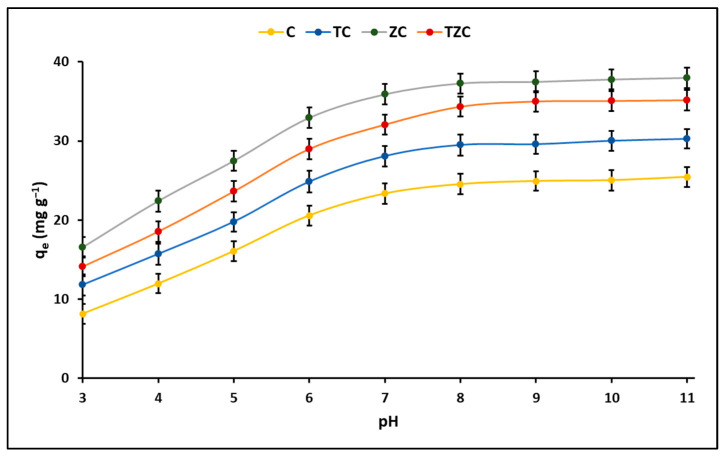
Effect of pH on the adsorption of cyanide species on extruded compounds. (Adsorbent = 200 mg L^−1^, KCN = 20 mg L^−1^, pH = 3–11).

The results of the cyanide adsorption experiment are presented in Figure 5, where the effect of the pH of the solution on the process is demonstrated. This figure shows that the total cyanide adsorption capacity (HCN + CN^−^) remained constant for all compounds within the pH range of 9 to 11, which is consistent with what was reported by other authors [39]. It is well known that the inorganic cyanides KCN and NaCN are weak acids and hydrolyze to form hydrocyanic acid (HCN), which has a pKa value of 9.4 [40]. However, several studies have reported that these cyanides can easily dissociate to form HCN and CN^−^ species at pH = 7.0 (pH < pKa) [41], suggesting that the high adsorption of cyanide species at neutral pH it is based on interactions with existing Lewis acid sites on the surface of the adsorbent [42,43]. Consequently, based on this evidence, the adsorption experiments were performed in this study at a pH of 7.0.

On the other hand, Figure 5 also indicates that compounds containing zeolite have a greater ability to remove cyanide species compared to compounds without zeolite. This effect is possibly due to the fact that zeolite has a greater specific surface area and a particular chemical composition, which allows a greater number of active sites available on the surface of the compounds to generate favorable kinetics [44].

#### 2.2.2. Adsorption Isotherm

This research analyzed the Langmuir, Freundlich, and Temkin isotherms as equilibrium models that are dependent on the initial concentration. The Langmuir isotherm provides a reliable theoretical basis for the process of adsorption on a uniform and homogeneous surface, where identical and specific sites are available for adsorption. The Langmuir model assumes that there is limited interaction between the molecules. On the other hand, the Freundlich equation is an empirical formula that does not assume uniformity in the energy of surface sites. It allows for an unlimited adsorption capacity and reflects a heterogeneous surface with an exponential distribution of active sites. The Temkin model assumes that the adsorption heat of all molecules decreases linearly with the increase in coverage of the adsorbent surface and that adsorption is characterized by a uniform distribution of binding energies up to a maximum binding energy. 

**Figure 6:**
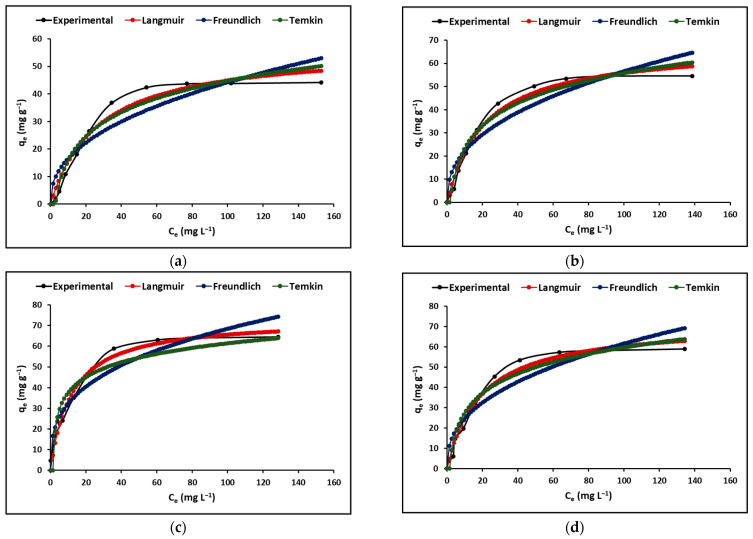
Absorption isotherms of the extruded compounds (**a**) C, (**b**) TC, (**c**) ZC, and (**d**) TZC. (Adsorbent = 200 mg L^−1^, KCN = 5–200 mg L^−1^, pH = 7.0 ± 0.1).

Figure 6 shows the cyanide adsorption isotherms of the C, TC, ZC, and TZC extruded samples. In this figure, it is evident that the Langmuir model is better than the Freundlich and Temkin models in describing the behavior of all samples.

Table 4 provides the estimated values of the Langmuir and Freundlich constants at various temperatures. The table reveals that the values of the R_L_ separation factor or equilibrium parameter ranged from 0 to 1, whereas the values of the coefficient n, which indicates the adsorption intensity, ranged from 1 to 10. These findings suggest that the adsorption of cyanide species on the surface of all samples was acceptable.

#### 2.2.3. Adsorption Thermodynamics

**Figure 7:**
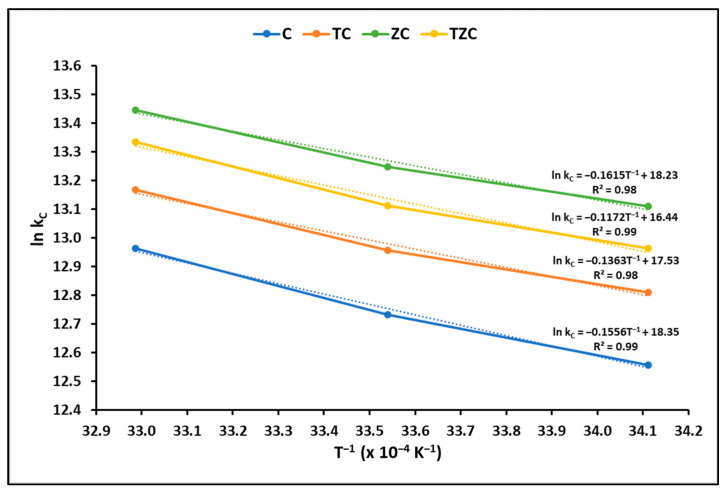
Thermodynamic study of cyanide adsorption on extruded compounds. (Adsorbent = 200 mg L^−1^, KCN = 20 mg L^−1^, pH = 7.0 ± 0.1).

The Gibbs free energy change (∆G°), enthalpy change (∆H°), and surface entropy change (∆S°) are thermodynamic parameters that offer information about the spontaneity and feasibility of a process. To determine these parameters, the equilibrium constant was evaluated at different temperatures, as shown in Figure 7.

The results of the thermodynamic parameters obtained in this study are shown in Table 5. On average, the Gibbs free energy (∆G°), enthalpy (ΔH°), and entropy (ΔS°) were calculated at −32.31 kJ mol^−1^, −27.14 kJ mol^−1^, and 0.20 kJ mol^−1^ K^−1^, respectively, which corroborates the spontaneous and exothermic behavior of the cyanide uptake by extruded samples.

#### 2.2.4. Adsorption Kinetics

**Figure 8:**
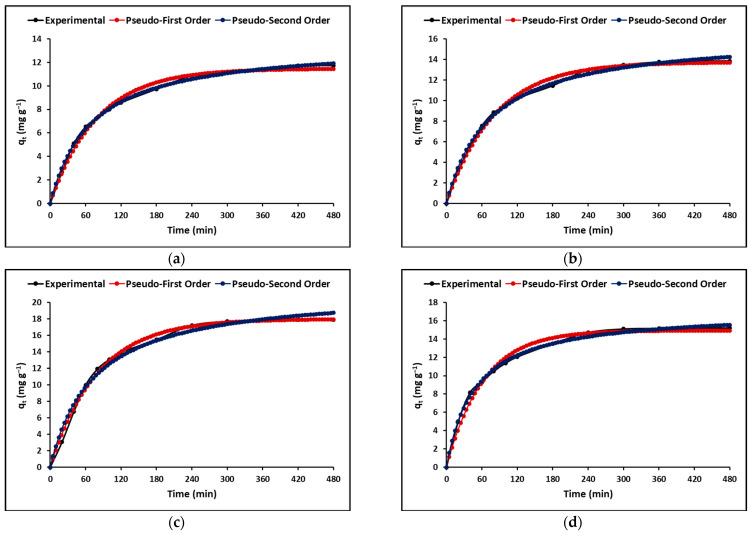
Adsorption kinetics of extruded compounds (**a**) C, (**b**) TC, (**c**) ZC, and (**d**) TZC. (Adsorbent = 200 mg L^−1^, KCN = 20 mg L^−1^, pH = 7.0 ± 0.1).

Determining the time-dependent process rate is crucial in the design and evaluation of materials for adsorption. Simplified models such as the Lagergren (pseudo-first-order) and Ho (pseudo-second-order) models are commonly used to describe adsorption kinetics. As illustrated in Figure 8, both models show a rapid initial adsorption phase followed by a plateau stage. Previous literature suggests that the pseudo-second-order model, as indicated in Table 6, exhibits a higher correlation coefficient than the pseudo-first-order model, indicating a chemisorption process [45].

**Figure 9:**
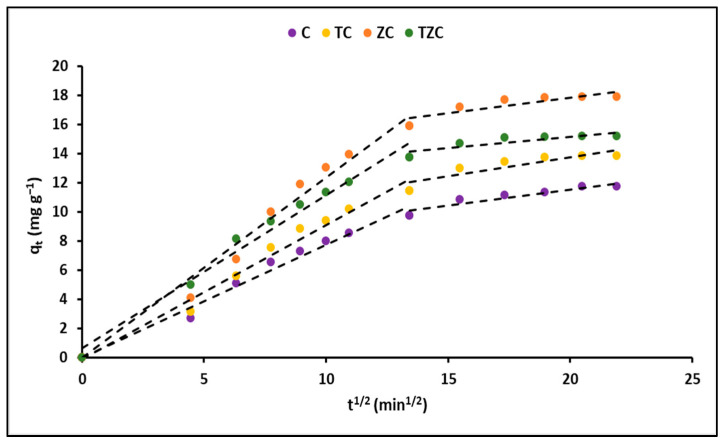
Intraparticle diffusion plots for cyanide removal by extruded compounds. (Adsorbent = 200 mg L^−1^, KCN = 20 mg L^−1^, pH = 7.0 ± 0.1).

Furthermore, the adsorption rate was explained using the intraparticle diffusion model, which takes into account the rate of transfer of cyanide species from the aqueous solution to the adsorption sites on the evaluated compounds. Figure 9 shows the variation of the q_t_ (mg g^−1^) curves as a function of time (t^1/2^) for the C, TC, ZC, and TZC compounds. 

Table 6 shows the total cyanide adsorption kinetic parameters estimated in this study for all samples.

### 2.3. Photodegradation Studies

**Figure 10:**
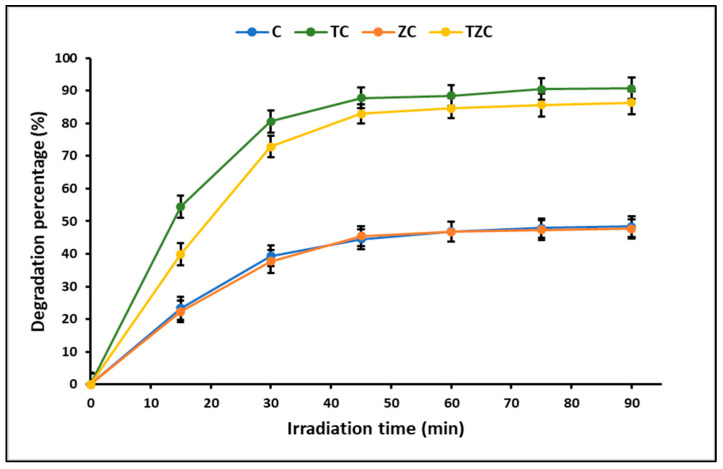
Photocatalytic cyanide degradation by the extruded compounds. (Catalyst = 200 mg L^−1^, KCN = 20 mg L^−1^, pH = 7.0 ± 0.1, λ = 310 nm).

ZnTiO_3_/TiO_2_ is a hybrid photocatalyst widely used to efficiently degrade organic compounds due to its high photooxidant capacity. In this study, the kinetics of cyanide photodegradation under ultraviolet radiation was estimated using the Langmuir–Hinshelwood equation. This equation showed a linear correlation between ln(C_0_/C_t_) and t, confirming that the cyanide species photodegradation reaction proceeds via a pseudo-first-order reaction. Apparent rate constants (k_app_) were calculated to be 0.020, 0.021, 0.024, and 0.025 min^−1^ for compounds C, ZC, TZC, and TC, respectively. These results are in agreement with those reported by other authors [5,46]. Figure 10 shows that the maximum percentage of cyanide degradation for all compounds is reached around the first 60 min, after which the photodegradation is apparently constant. According to the literature, there is a limit to the efficacy of photocatalysis for the complete degradation of some pollutants [25]. In fact, in this study, the maximum efficiency was accomplished by TC (90.7%), followed by TZC (86.3%), C (48.4%), and ZC (47.7%). 

### 2.4. Total Efficiency and Reuse of Compounds

**Figure 11:**
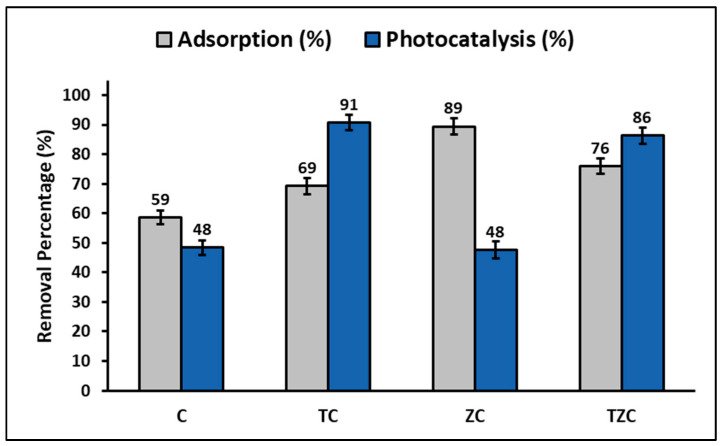
Percentage of cyanide adsorbed and photodegrade by the extruded compounds.

The percentage of cyanide adsorbed and photodegraded by compounds C, TC, ZT, and TZC is shown comparatively in Figure 11. In this figure, it can be seen that the efficiency of the adsorption process decreases in the following order: ZC > TZC > TC > C, while the efficiency of the photocatalytic process decreases in the following order: TC > TZC > C > ZC.

**Figure 12:**
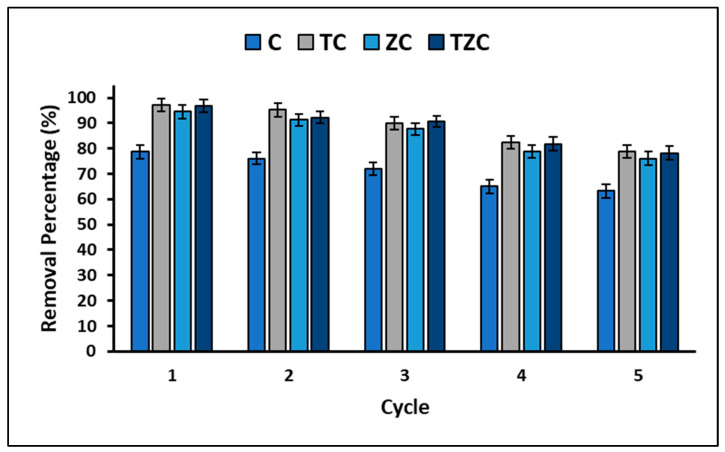
Extruded compounds reuse experiment.

Finally, the stability and reuse of the materials used for their adsorbent and photocatalytic properties are critical factors to consider for their extensive implementation. To address this, the study conducted experiments to examine the reuse potential of the compounds during several successive cycles of cyanide removal. The results obtained are represented in Figure 12, which illustrates the removal efficiency of the synthesized compounds over the five cycles.

## 3. Discussion

### 3.1. Characterization of the Compounds

In this study, XRD was employed to identify the crystalline structure of the clay, zeolite, mixed oxide ZnTiO_3_/TiO_2_, and TZC composite. Figure 1 depicts a comparison of the diffraction patterns of the zeolite with the precursor clay, indicating the disappearance of reflections from the mineralogical phases in the clay. The zeolite diffraction pattern exhibited the appearance of peaks that correspond to the FAU phase, along with some impurities from the LTA and NaP1 zeolite phases. The existence of these zeolitic phases demonstrated the transformation of the clay, which occurs initially with the generation of an amorphous material, followed by the co-crystallization of FAU, LTA, and NaP1 zeolitic phases. These outcomes are consistent with the metastable nature of the zeolite phases obtained. As per the literature, the mixture of the metastable phases obtained could have been facilitated by the differences in the Si/Al ratio and the effect of the cations present in the original clay [47,48,49] The diffraction patterns were used to determine the percentage of crystallinity of the zeolitic material through Equation (1), which was found to be 85%. Generally, the formation of the amorphous phase (15%) could be associated with the presence of geopolymers [50]. Specifically, geopolymers can be recognized as the amorphous equivalent of crystalline aluminosilicate structures, with the same chemical composition as zeolites, but showing a disordered structure, unlike the ordered structure of zeolites [51,52,53]. In regards to the results, the diffraction pattern illustrated that the clay was made up of montmorillonite (M), albite (L), cristobalite (C), quartz (Q), and heulandite (H). Furthermore, Figure 1 presents the diffraction pattern of the ZnTiO_3_/TiO_2_ heterostructure. In this study, the hybrid photocatalyst was synthesized at 500 °C with a ZnO:TiO_2_ molar ratio of 1:3, obtaining a nanoparticulate material with a composition of 53% ZnTiO_3_ phase and 47% TiO_2_ phase (anatase phase). In previous studies we reported the synthesis of the hybrid photocatalyst using different ZnO:TiO_2_ molar ratios as well as various calcination temperatures [33]; however, the highest percentage of crystallinity and purity of the desired phases of ZnTiO_3_ and TiO_2_ (anatase) were obtained with the conditions that we present in this study.

When comparing in Figure 1 the diffraction patterns of the TZC composite with those of its individual components, no significant changes are observed in the main diffraction peaks of the zeolite or the ZnTiO_3_/TiO_2_ mixed oxide. The stability of these crystalline phases in the extruded composite was probably due to the fact that the temperature used for the respective calcination (500 °C) was below the thermal stability of the Faujasite-type zeolite [54] and the mixed oxide ZnTiO_3_/TiO_2_ [33]. Consequently, in this study, it was possible to obtain a material with adsorbent and photocatalytic properties that is also mechanically and chemically stable.

Samples of clay, faujasite zeolite, ZnTiO_3_/TiO_2_ mixed oxide, and TZC composite were also characterized in terms of their morphology and chemical composition by SEM-EDX analysis. The SEM micrographs shown in Figure 2a–c revealed that the clay, zeolite, and mixed oxide structures, respectively, are similar to those previously reported for these compounds by different authors [55,56,57]. On the other hand, the SEM photomicrograph presented in Figure 2d shows an enlarged view of the morphology of the TZC compound. In this figure, it can be seen cubic particles of faujasite zeolite that have a more or less uniform size, particles of clayey material of different sizes and shapes, as well as tiny, nearly spherical photocatalyst granules. In general, Figure 2d shows a heterogeneous mixture of particles that make up the TZC composite. 

From the EDX results shown in Figure 3, the decrease in the percentage by weight of the elements of the starting clay is observed, probably due to dilution effects when adding new components, such as alumina and sodium hydroxide, in the process of zeolitization [58]. In fact, the percentage by weight of Na^+^ increases significantly due to the incorporation of NaOH as a mineralizing agent for the synthesis of zeolites [59,60]. This increase was a result of the capture of Na^+^ ions by the zeolitic structure to neutralize the negative charge of aluminum in the zeolite and/or geopolymer formed [61]. Figure 3d shows the elemental composition of the TZC composite, where the presence of the constituent elements of the starting compounds is evident. Additionally, the EDX analysis confirmed the presence of several cations (Ca, K, Fe, Na) in the clay, zeolite and TZC composite, which may provide adsorbent properties to the materials for enhancing the removal of cyanide species through cation exchange.

On the other hand, the reduction in the surface area of the extruded adsorbents following calcination observed in this study is mainly due to the removal of certain components from the internal surface of the clay, such as physisorbed water and weakly bonded superficial hydroxyl groups in the clay structure. This process generates more space within the large pores of the clay structure, which promotes the contraction of the structure and consequently leads to a decrease in internal surface area [62]. The clay utilized in this study as an inorganic binder contributed to the strength and durability of the extruded compounds, but it also seems to have contributed to the blockage of the pores of the active porous component, namely zeolite, leading to a lower surface area compared to zeolite alone. However, the inclusion of zeolite as a porous material in the extruded compounds increased their adsorption capacity for cyanide species in the aqueous solution.

Finally, even though powders usually exhibit higher specific surface areas, in this research, the extruded adsorbents were preferred for the adsorption of cyanide species because of their satisfactory mechanical and chemical durability, which allowed for easy retrieval at the conclusion of the process and their reuse after multiple treatment cycles.

### 3.2. Adsorption Studies

A preliminary batch adsorption test of cyanide species from an aqueous solution was performed to investigate the adsorption efficiency of clay, zeolite, ZnTiO_3_/TiO_2,_ and composites that were used in powder and/or extrudate form. Figure 4 shows that zeolites had the highest adsorption capacity. This great adsorption capacity was because zeolites have a porous structure and, therefore, a higher specific surface than clays, so they can easily host different molecules on their surface [63,64]. On the other hand, the extrudates showed a lower specific surface area but were also effective in removing cyanide species from the aqueous solution, probably through other mechanisms, including electrostatic interaction, chemical reactions such as complexation, or ion exchange between sorbent and cyanide species [65]. Consequently, despite the reduction in specific surface area in the extrudates, the surface chemistry of these materials was also an important factor controlling cyanide species adsorption. 

It is widely known that the adsorption process occurs by different mechanisms; therefore, several batch adsorption experiments of cyanide species from an aqueous solution were developed in this study to investigate the performance of extruded materials. The experiments were carried out by varying the following parameters: the initial pH of the cyanide solution, the initial cyanide concentration, the temperature, and the contact time.

#### 3.2.1. Effect of pH

It is well known that the pH of a solution significantly influences the surface charge of the adsorbents and the ionization and speciation of the adsorbate. Consequently, for a better explanation of the adsorption mechanism of cyanide species on the surfaces of compounds C, TC, ZT, and TZC, it is essential to consider the pKa of cyanide as well as the pH_PZC_ of the compounds. According to the literature, cyanide has a pKa value of 9.4 [40]. Therefore, at pH values below the pKa, cyanide is mostly in its molecular form (HCN), while at pH values above the pKa, cyanide is mainly dissociated in its ionic form (CN^−^ + H^+^) [66]. Regarding the zero point of charge values (pH_PZC_) of the tested compounds, Table 3 provides the respective information. In general, at pH levels above pH_PZC_, an increase in the number of negatively charged groups occurs, while at pH levels below pH_PZC_, an increase in the number of positively charged groups occurs. 

The information obtained in this study through XRD, XRF, and EDX analysis for the clay and zeolite suggests that both materials have chemical structures formed by channels that contain various cations (Na^+^, K^+^, Ca^2+^, Fe^3+^). Therefore, the adsorption of organic molecules, including cyanide species, could take place both at the edges of the crystal structure and in structural channels. The clay and zeolite used in this study are in their sodium form, which could result in the release of alkaline cations, such as Na^+^, in a basic medium to react with OH^−^ and to form sodium hydroxide (NaOH). This process could alkalinize the medium and lead to pH values close to the pKa = 9.4 of cyanide. According to the literature, ion dissociation could be incomplete and, at pH values ≈ 8, (pH < pKa) C−HCN or Z−HCN hybrid systems could be formed, resulting in partial adsorption of the HCN species on both the clay as on the zeolite. In this study, all cyanide species removal experiments were performed keeping the pH value = 7, since a minimal increase in the adsorption of cyanide species in the solution was observed at pH values higher than 8.

As previously mentioned, the presence of the CN^−^ anion becomes predominant above the pKa value, whereas, below this value, HCN occurs in molecular form. Although the CN^−^ anion is negatively charged and should not normally be able to enter positively charged structural channels in clay or zeolite, its small size increases the possibility that it will enter negatively charged channels. The same is true for undissociated HCN molecules. Although at neutral or acidic pH HCN does not dissociate and therefore has no net charge, its small size may allow it to enter structural channels. At very acidic pH, HCN is protonated according to its pKa species distribution, increasing its degree of adsorption within negatively charged channels. However, pH alone does not fully explain the differences in the amount of adsorption of cyanide species on clay and zeolite, since other factors, such as van der Waals forces, ionic strength of the solution, the size and shape of the molecules, as well as coadsorption and steric hindrance, are also important in determining the degree of adsorption. At pH values ≥ pKa, most species are neutral or negatively charged and do not adsorb into negatively charged structural channels, but a small amount of adsorption can still be observed. At pH 10, the species are negatively charged and do not show clear adsorption of cyanide species.

The ZnTiO_3_/TiO_2_ mixed oxide immobilized on the clay/zeolite has a pH-dependent surface charge due to the protonation-deprotonation equilibrium of the hydroxyl groups on the surface [67]. Previous studies have shown that titanium oxides can act as weak Brönsted acids and form surface hydroxyl groups (Ti−OH) upon hydration [68]. These hydroxyl groups can participate in proton dissociation and association reactions, generating a pH-dependent surface charge [69]. Studies indicate that at pH < pHPZC, the specific adsorption of HCN species on the OH groups of hydrated titanium oxide nanoparticles occurs through the formation of H–N polar covalent bonds, such as TiOH–NCH or TiOH_2_^+^–NCH [66].

When the pH is less than 7.0, the charge on the surface of the ZnTiO_3_/TiO_2_ nanoparticles is positive, and the dissociation of HCN is minimal, so it is unlikely that electrostatic forces of attraction between a charged surface and a charged surface will occur. A neutral molecule, especially at very low pH values. However, as the pH increases and a pH of 7.0 are reached, the negative charge on the surface of the nanoparticles increases, leading to an increase in electrostatic attractive forces and, thus, an increase in the adsorption of HCN species on the negative surface of ZnTiO_3_/TiO_2_ nanoparticles in the pH range between 7.0 and 9.4. When the pH is higher than 9.4, the proportion of CN^−^ ions in the solution increases, but at the same time, the surface of the ZnTiO_3_/TiO_2_ nanoparticles becomes more negative because the pH is higher than the point of charge, zero (pH_PZC_), which generates repulsive forces between the negative surface sites of the nanoparticles and the negative CN^−^ ions. This results in a reduction in the adsorption capacity of these ions at very high pH values, as shown in Figure 5. It is expected that the specific adsorption of the HCN species on the OH groups present on the surface of the nanoparticles of hydrated titanium oxides is carried out through the formation of polar H–N covalent bonds (i.e., TiOH–NCH or TiOH_2_^+^–NCH) at pH < pH_PZC_.

The relationship between the pH of the solution and the adsorption capacity of the synthesized compounds suggests that, in this study, the total cyanide adsorption could have been mainly driven by electrostatic interactions, although the possibility of other types of interactions, such as van der Waals and/or specific interactions, is not ruled out. It is suggested that the adsorption of CN^−^ species on the surface of the compounds could occur predominantly by chemical adsorption, although the presence of physical adsorption is not completely excluded. These results are consistent with those reported in the literature. The presence of active cationic sites (Na^+^, K^+^, Ca^2+^, Al^3+^, Fe^3+^) on the surface of the compounds contributes to the formation of Lewis acid sites, which could enhance cyanide adsorption on the surface of the materials [70] and provide catalytic stability in an aqueous reaction system [71].

#### 3.2.2. Adsorption Isotherm

Figure 6 displays the adsorption isotherms of all the compounds examined in this study. It is observed that at low concentrations of cyanide species (HCN + CN^−^), the adsorption rate increases, while at high concentrations, it reaches a maximum level and stabilizes. This could be attributed to the excessive presence of cyanide species competing for the available active sites on the adsorbent compounds’ surface. The results suggest that the initial concentration of cyanide species in solution creates a significant driving force to allow these species to move from the liquid phase to the nanoparticle surface [72].

The Langmuir, Freundlich, and Temkin isotherm models were applied in this study to analyze the adsorption data acquired for the compounds. The corresponding parameters for the experimental data are presented in Table 4. The values of the correlation coefficient (R^2^) reveal that the Langmuir model is the most appropriate to describe the equilibrium adsorption behavior, indicating that monolayer adsorption of cyanide species on the synthesized compounds occurs as a phenomenon of electrostatic attraction. This attraction takes place in regions with homogeneous surfaces where the strongest binding sites are initially filled, and as the saturation level rises, the binding strength decreases [73]. Finally, the Langmuir and Freundlich constants (R_L_ and n) shown in Table 4 confirm that the synthesized compounds have a favorable adsorption capacity for cyanide species.

#### 3.2.3. Adsorption Thermodynamics

In this research, various thermodynamic parameters were analyzed to determine how effective and feasible it is to adsorb cyanide species on the synthesized compounds. Table 5 illustrates the thermodynamic parameters obtained, which include ∆G°, ∆H°, and ∆S°. The Gibbs free energy (∆G°) indicates the level of the spontaneity of the process, with negative values suggesting higher adsorption favorability. Similarly, negative enthalpy values (ΔH°) show that the adsorption is exothermic, supporting the notion that physisorption was mainly responsible for cyanide adsorption on the extruded samples and that the adsorption complexes produced are energetically stable. Positive entropy values (ΔS°) indicate that the randomness at the solution–solid interface increased during the adsorption process. These results are consistent with previous studies reported by other authors [74].

#### 3.2.4. Adsorption Kinetics 

The use of kinetic models in adsorption studies allows for determining the time required for complete removal of a chemical species. In this study, the experimental data obtained for the adsorption of cyanide species on the synthesized compounds were fitted with pseudo-first-order and pseudo-second-order kinetic models, as shown in Figure 8. The results indicated that the concentration of cyanide species decreased rapidly in the initial stages of the adsorption process and became constant after approximately 90 min. This rapid adsorption can be attributed to the high concentration gradient and the availability of vacant adsorption sites. The kinetic parameters obtained from the fitting of the data to the models are presented in Table 6. The correlation coefficient (R^2^) indicated that the experimental data were better described by the pseudo-second-order model, suggesting chemical adsorption of cyanide species on the surface of the synthesized compounds [75]. Additionally, the intraparticle diffusion model was used to analyze the mass transfer process. Figure 9 shows that the adsorption of total cyanide occurs in two stages, with the initial stage corresponding to the diffusion of cyanide particles through the stationary film that surrounds each adsorbent compound. The second stage corresponds to intraparticle mass transfer, where the cyanide particles diffuse through the pores of the adsorbent compounds. The kinetic parameters obtained from the fitting of the data to the models are presented in Table 6, with relatively high values of A suggesting that surface adsorption could be the rate-limiting step for all synthesized compounds [73]. 

### 3.3. Photodegradation Studies

This study demonstrated that the TC and TZC compounds were the most effective in photodegrading total cyanide from aqueous solutions. Both TC and ZTC composite materials have been shown to be effective in cyanide removal in this study, although each has specific strengths and weaknesses. On the one hand, the TC composite is more efficient as a photocatalyst than the ZTC due to the higher content of the ZnTiO_3_/TiO_2_ hybrid semiconductor, which allows it to photodegrade cyanide by generating electron-hole pairs under the action of light. However, the TC composite has a lower adsorption capacity for cyanide species than ZTC, which means that it cannot efficiently capture these species for subsequent decomposition. On the other hand, the ZTC composite is a better cyanide adsorbent than TC due to its zeolite content, which gives it a greater porous surface and availability of active sites to form chemical bonds with cyanide species. However, ZTC is less photoactive than TC, which means that it has a limited ability to degrade cyanide species under the action of light. The combination of adsorption and photocatalysis is an important strategy for efficient cyanide removal because both techniques work in synergy to improve the efficiency of cyanide removal. Adsorption can be used to capture cyanide species present in the water, and then photocatalysis can be applied to degrade the adsorbed cyanide species. In this process, adsorption provides an efficient way of concentrating cyanide species in one location, increasing the efficiency of photocatalysis. Consequently, the combination of both mechanisms, adsorption and photocatalysis, is an effective strategy for cyanide removal, and the choice of the specific material, TC or ZTC, will depend on the specific needs of the application.

Furthermore, in the present study, the clay, in addition to its adsorbent capacity, also demonstrated some photocatalytic activity since this porous material contains various metal oxides, including SiO_2_ and Fe_2_O_3_. The presence of SiO_2_ in the clay would provide an adsorbent surface for contaminants, while Fe_2_O_3_ would provide some photocatalytic activity to the mineral. In fact, Fe_2_O_3_ is a transition metal oxide formed by different crystal structures, such as hematite (α-Fe_2_O_3_), among others. Figure 1 shows the XRD pattern of the clay, where some peaks corresponding to the hematite phase can be distinguished. According to the literature, α-Fe_2_O_3_ hematite has shown great potential in various photocatalytic applications due to its low cost, non-toxicity, strong antiferromagnetic properties, excellent stability, easy recovery, as well as appropriate band gap energy (E_g_ = 2.0–2.2 eV) to take advantage of a broad solar spectrum. In addition, hematite is an economical material due to its abundance in nature, and it also has a resistance to corrosion in both alkaline and acidic media [76,77,78,79].

On the other hand, it is widely known that the heterojunction of two efficient semiconductors can lower the bandgap energy of the hybrid semiconductor, resulting in better photoactivity than that of the individual semiconductors. Indeed, the bandgap energy plays an important role in the photoactivity of semiconductors since it determines the recombination rate of electron/hole pairs. Likewise, other factors that can affect the recombination rate include carrier concentration and carrier mobility, as well as semiconductor structure [80]. Therefore, the results of this study suggest that the immobilization of the ZnTiO_3_/TiO_2_ hybrid semiconductor on the porous support improved the photoactivity of the TC and TZC compounds by modifying the recombination rate of the electron/hole pairs. These results are supported by a previous study where we demonstrated that the ZnTiO_3_/TiO_2_ hybrid semiconductor had lower bandgap energy than the individual semiconductors [33], which suggests that given the smaller separation between the valence (VB) and conduction (CB) bands, the hybrid photocatalyst could easily transfer photoinduced electrons from the bulk to the surface and be more photoactive than individual semiconductors [81]. According to the literature, due to this electron transfer, a series of sequential reactions take place near the surface of the hybrid photocatalyst, generating reactive oxygen species (ROS), including radical superoxide anions (O_2_˙^−^), singlet oxygen (O_2_˙), hydrogen peroxide (H_2_O_2_), and hydroxyl radicals (OH˙). These species are highly reactive and can oxidize and break down cyanide into less toxic compounds such as cyanate, carbonate, and nitrate [82,83,84,85,86]. In a previous study, we reported that hydroxyl radicals are the most reactive species generated by titanium oxide. These radicals can attack cyanide in multiple positions, oxidizing it and breaking it down into less toxic compounds [87]. Therefore, based on the results of the previous studies and the current one, it is suggested that the incorporation of the ZnTiO_3_/TiO_2_ hybrid photocatalyst in porous matrices such as zeolite and clay is an effective and sustainable alternative for the removal of cyanide species from aqueous systems.

### 3.4. Total Efficiency and Reuse of the Compounds

This study found that C and ZC compounds are more efficient in cyanide species adsorption, while TC and TZC compounds are more efficient in cyanide species photodegradation under the given test conditions, as shown in Figure 11. The adsorption process can be influenced by several factors, such as the properties of the sorbent and sorbate and the conditions of the solution. The study showed that the pH of the solution plays an important role in the adsorption capacity of cyanide species in the synthesized compounds. For this reason, to determine the efficiency of the compounds for cyanide removal, the reaction system was maintained at pH = 7.0 ± 0.1 by adding 0.1 M solutions of hydrochloric acid (HCl) or sodium hydroxide (NaOH). Throughout the experiment, the pH of the solution was controlled, both in the adsorption and photodegradation tests, since it is widely known that an increase/decrease in pH can occur in these processes due to the release of anionic and cationic ions toward the aqueous solution [88]. Figure 5 indicates that the maximum adsorption capacity occurs at pH values greater than 9. However, since the adsorption tests were performed at pH 7, the relatively lower adsorption percentages at this pH can be attributed to the limited electrostatic attraction between cyanide species and the surfaces of synthesized compounds, whose pH_ZPC_ values (in the range of 6.8–7.8) are lower than the pKa of cyanide species (9.4).

The study also demonstrated the presence of cationic elements in the clay and zeolite, which could generate Lewis acid sites on the surface of the synthesized compounds, contributing to improving the adsorption capacity of cyanide species or favoring cation exchange. In fact, clays and zeolites are aluminum silicates with a large surface area, functional channels, and adjustable pore size [38]. On the surface of these materials, there are Lewis acid sites that can be metal ions, such as Na^+^, Fe^3+^, or Al^3+^, or chemical species containing a partially positively charged carbon atom [89]. These Lewis acids can accept an electron pair from a negatively charged species, making them ideal for the storage of corrosive chemicals, such as cyanide [90]. Thus, when the adsorbent is in contact with a solution containing cyanide, the cyanide ions present in the solution can interact with Lewis acid sites on the surface of the adsorbent by nucleophilic addition [66]. Once the cyanide is adsorbed on the Lewis acid sites, a cyanide–Lewis acid complex is formed. This complex is stable and can be recovered from the adsorbent by elution processes using appropriate solutions [90]. It is important to note that cyanide adsorption on Lewis acid sites on the adsorbent surface can be influenced by several factors, such as solution pH, cyanide concentration, nature and number of acid sites Lewis on the adsorbent, and the presence of other chemical species in the solution. Therefore, it is necessary to carefully consider these factors to optimize the adsorption process and achieve effective and efficient removal of cyanide from the solution [91].

In addition, the addition of the ZnTiO_3_/TiO_2_ hybrid semiconductor in the TC and TZC compounds improved their efficiency in removing cyanide species by combining adsorption and photocatalysis processes. This is because the cyanide species that initially adsorbed and accumulated on the nanoparticle surface at the beginning of the photocatalytic degradation are the first to degrade under irradiated light. Therefore, the constant migration and the successive photocatalytic oxidation on the surface of the compound contribute to improving the removal efficiency at the solid–liquid interface, generating a concentration gradient that acts as the main driver of the removal process of cyanide species in the aqueous solution. Consequently, this study suggests that the removal of cyanide species is due to the cooperative effect between adsorption and photocatalysis processes. The synthesized compounds exhibit effective performance in cyanide removal from aqueous systems by coupling “adsorption-photodegradation” processes [68].

On the other hand, it is widely recognized that the useful life and potential applications of a material are closely linked to its structural and chemical stability. In this study, the efficacy of the reuse of extruded compounds was evaluated through five consecutive treatment cycles, with the aim of estimating their efficacy. Figure 12 shows the results of the experiment, which indicate that the percentage of cyanide removal decreases with each cycle. Despite this, after five cycles, the loss of cyanide removal capacity for compound C did not exceed 15.44%, while the average for compounds ZC, TC, and TZC was 18.45%. The compounds were regenerated after each cycle by washing with a saturated sodium chloride solution [74]. The observed decreased efficacy may be due to the chemical adsorption of cyanide species onto the surface of compounds, which reduces the availability of active sites. However, XRF analysis confirmed that there were no significant changes in the original composition of the compounds after the fifth cycle. Therefore, compounds C, TC, ZC, and TZC are chemically stable and maintain adequate activity for up to five treatment cycles, effectively removing cyanide species by adsorption and photocatalysis. Regarding the mechanical stability of the tested compounds, this is directly related to their useful life since poor mechanical stability can cause disintegration in the reaction solution, which would lead to loss of activity and contamination. It is widely known that the mechanical stability of structured materials is correlated with the calcination temperature [92]; thus, higher temperatures achieve better stability. However, there is an optimum temperature to achieve maximum mechanical stability. In this study, a maximum calcination temperature of 500 °C was used to avoid crystalline phase change in the synthesized compounds. In this way, it was found that the extruded materials were mechanically stable and demonstrated effectiveness in the removal of cyanide species, which suggests their effective application in wastewater remediation processes.

In order to validate the efficiency of the prepared materials and demonstrate the contribution of this study to the field, the results obtained were compared with those reported by other authors. Therefore, Table 7 presents a comparison between the maximum adsorption capacity (mg g^−1^) of the compounds under investigation and some other adsorbents utilized for removing cyanide from aqueous solutions. Likewise, Table 8 provides a comparison of the photodegradation efficiency (%) between the compounds under investigation and other photocatalysts employed for eliminating cyanide from aqueous solutions.

The cyanide adsorption data listed in Table 7 show that among all the materials prepared in this study, the ZC composite (zeolite + clay) presents the highest adsorption value of cyanide species. When compared with other materials reported in the literature, the table shows that Clay-K presents the highest adsorption value, followed by the metal oxides ZnO, NiO, and ZnO-NiO. On the contrary, the LTA zeolite modified with HDMTMAB presents the lowest adsorption value, while the Ce/ZnTiO_3_, La/ZnTiO_3_, and TiO_2_/Fe_2_O_3_ materials present moderate adsorption values of cyanide species. Evidence from this study shows that the combination of clay, zeolite, and photocatalyst (TZC) appears to have a cyanide adsorption capacity comparable to clay with zeolite (ZC) and superior to clays with photocatalyst (TC) and clay alone (C). This suggests that the zeolite significantly improves the adsorption capacity of the clay for cyanide, while the photocatalyst alone does not have a significant impact. 

The cyanide photodegradation data listed in Table 8 show that the C, ZC, and TZC composites have a relatively low efficiency compared to other materials such as La/ZnTiO_3_, Ce/ZnTiO_3_, La/TiO_2_, Ce/TiO_2_, TiO_2_/Fe_2_O_3_/PAC, BFS, Cts-Ag, and TC. The presence of photocatalysts such as TiO_2_ and Fe_2_O_3_ significantly improves cyanide degradation efficiency in various compound materials, such as TiO_2_/Fe_2_O_3_/zeolite and TiO_2_/Fe_2_O_3_/PAC. Furthermore, it is observed that the doping of semiconductors with rare earth elements also has a positive effect on cyanide photodegradation efficiency. Among the materials prepared in this study, the TC composite, which is clay with a photocatalyst, shows high efficiency in cyanide photodegradation. On the other hand, the ZC material, which is clay with zeolite, has a relatively low efficiency, which suggests that zeolite alone is not sufficient for suitable cyanide photodegradation.

From the adsorption and photodegradation results presented in this investigation and comparing them with those cited in the literature, it can be inferred that the composites C, TC, ZC, and TZC are efficient in removing cyanide species from aqueous solutions. Furthermore, the findings suggest that the removal of cyanide was controlled by a combination of electrostatic interactions, formation of complexes through coordinate covalent bonds, and photo-oxidation that occurs on the surface of synthesized composites upon exposure to UV radiation. In addition, it is important to point out that in this study, the composites were used in the form of extrudates, so in addition to their efficacy for cyanide removal, they also have advantages in terms of handling and practical applications. Compared to powdered materials, extrudates are easier to handle and have better mechanical strength, allowing them to better withstand abrasion and wear during use. They are also easier to package and transport, reducing the costs and risks associated with handling powdered materials. Another advantage is that extrudates can have greater durability and stability in the environment. Powdered materials are more susceptible to degradation and can dissolve or disperse in water or air, limiting their effectiveness and potentially causing environmental problems. On the other hand, materials in the form of extrudates have a more solid and compact structure, which allows them to better resist adverse environmental conditions and maintain their effectiveness for longer periods of time.

## 4. Materials and Methods

### 4.1. Materials

All reagents were of analytical grade and were applied in this study without further purification: titanium (IV) isopropoxide (Ti(OC_3_H_7_)_4_, Sigma Aldrich, St. Louis, MO, USA, 98.0%), zinc acetate ((CH_3_CO_2_)_2_Zn, Sigma Aldrich, St. Louis, MO, USA, 99.99%), isopropyl alcohol (C_3_H_8_O, Sigma Aldrich, St. Louis, MO, USA, ≥99.5%), hydrochloric acid (HCl, Sigma Aldrich, St. Louis, MO, USA, 37.0%), sodium hydroxide (NaOH, Sigma Aldrich, St. Louis, MO, USA, ≥85.0%), potassium cyanide (KCN, Sigma Aldrich, St. Louis, MO, USA, ≥97.0%), picric acid ((O_2_N)_3_C_6_H_2_OH, Sigma Aldrich, St. Louis, MO, USA, ≥99.0%), sodium carbonate (Na_2_CO_3_, Sigma Aldrich, St. Louis, MO, USA, ≥99.0%), hydrogen peroxide (H_2_O_2_, Sigma Aldrich, St. Louis, MO, USA, 35%), silver nitrate (AgNO_3_, Sigma Aldrich, St. Louis, MO, USA, >99.8%), nitric acid (HNO_3_, Sigma Aldrich, St. Louis, MO, USA, 69%), sodium metasilicate nonahydrate (Na_2_O_3_Si·9H_2_O, Sigma Aldrich, St. Louis, MO, USA, ≥98.0%), sodium aluminate (Sigma Aldrich, St. Louis, MO, USA, ≥98.0%), Al(Al_2_O_3_): 50–56%, Na(as Na_2_O): 37–45%).

### 4.2. Clay Purification

The clay sample was collected in the Province of Loja in southern Ecuador (3°59′0″ S, 79°21′0″ W, 1238 m.s.l.). The clay sample underwent grinding and sieving until it reached a size of 200-mesh (0.074 mm). To remove calcium and magnesium carbonates, hydrochloric acid (0.1 N) was added in a proportion of 10 mL g^−1^. Organic matter within the clay sample was eliminated by the addition of H_2_O_2_ (33%) in a ratio of 10 mL g^−1^ while stirring for 2 h at room temperature. The clay was then centrifuged, and the product was washed with distilled water to remove Cl^−^ ions. The removal of these ions was confirmed with an AgNO_3_ test. To activate the clay adsorption sites, nitric acid (0.8 N) was added in a ratio of 10 mL g^−1^. After activation, the clay sample was centrifuged, washed with distilled water, and dried at 60 °C for 24 h.

### 4.3. Synthesis of ZnTiO_3_/TiO_2_ Nanoparticles

The synthesis of ZnTiO_3_/TiO_2_ nanoparticles was carried out following the sol-gel method described in previous studies [33]. To begin, titanium isopropoxide (TiPO) in isopropyl alcohol (iPrOH) (70% *v*/*v*) was dispersed at room temperature. Then, an aqueous solution consisting of Zn(OAc)_2_/water/iPrOH was slowly added to the mixture using ZnO/TiO_2_ at a 1:3 molar ratio. The amount of water added was equivalent to the stoichiometric amount required for hydrolyzing the TIPO molecules, maintaining an iPrOH/water ratio of 50% *v*/*v*. The synthesis process was conducted at room temperature, and the reaction system was stirred for an additional 30 min after the formation of a precipitate. The precipitate was dried at 60 °C for 24 h and calcined at 500 °C for 4 h. Finally, the product was cooled to room temperature.

### 4.4. Synthesis of Zeolite from Ecuadorian Clay

The zeolite synthesis conditions were established based on previous research [101]. The initial process included mixing 20 g of clay with 25 g of NaOH and dissolving them in 50 mL of water to form a homogeneous slurry. Then, the clay-NaOH slurry was calcined at 800 °C for 5 h, followed by griding and suspension in 128 mL of water. To synthesize the zeolite, sodium metasilicate nonahydrate, sodium aluminate, and sodium hydroxide were added to achieve the desired composition: SiO_2_/Al_2_O_3_ = 4.0, Na_2_O/SiO_2_ = 1.65, H_2_O/Na_2_O = 40. The mixture was stirred at room temperature for 1 h until homogeneous. Then, it was left to age for 24 h at room temperature. The hydrothermal treatment was carried out by heating the covered containers to 90 °C for 24 h. The solid product was filtered, washed with water to remove excess alkali, and dried at 90 °C overnight. The resulting product was primarily Na-FAU type zeolite with minor amounts of Na-P1 type zeolite.

### 4.5. Preparation of Extruded Samples

To assess the solid materials, cylindrical extrudates with dimensions of approximately 1.0 cm in length and 0.2 cm in diameter were fabricated. These were prepared by mixing ZnTiO_3_/TiO_2_ nanoparticles with zeolite and precursor clay in a ratio of 30:30:40, respectively. In addition, extrudates were fabricated by mixing zeolite with clay in a 60:40 ratio, ZnTiO_3_/TiO_2_ nanoparticles with clay in a 60:40 ratio, and using only clay without any other materials. Approximately 35% water was added to each mixture to form a paste with suitable plasticity, and they were extruded using a 2.5 mm diameter syringe. The extrudates were dried at 90 °C for 2 h and then calcined at 500 °C for 8 h. The formulation of the different composites was established based on physical stability tests, in which the weight variation (disintegration) of the extrudates was evaluated after 24 h of agitation in an aqueous medium. It is important to note that the presence of clay is crucial for the formation of extrudates since composites prepared only with the semiconductor and the zeolite easily disintegrate. The extruded composites were labeled as follows: C (clay only), ZC (zeolite and clay), TC (ZnTiO_3_/TiO_2_ and clay), and TZC (ZnTiO_3_/TiO_2_, zeolite and clay).

### 4.6. Characterization 

X-ray diffraction (XRD) measurements were conducted using a Bruker-AXS D8-Discover diffractometer (Bruker AXS, Karlsruhe, Germany), which operated at 40 kV and 40 mA to generate Cu Kα radiation (1.5406 Å). The data were collected within the 2θ range from 5 to 70°, and the ICDD database (International Center for Diffraction Data, version 2018) and COD database (Crystallography Open Database, version 2021) were employed to recognize the crystalline phases. X-Ray fluorescence (XRF) measurements were recorded in a Bruker S1 Turbo SDR portable spectrometer (Bruker Handheld LLC, Kennewick, WA, USA) using the Mining Light Elements measurement method. In order to determine the specific surface area (SSA) of the solids (m^2^/g), nitrogen adsorption was carried out at the temperature of liquid nitrogen (−196 °C) using a 30% gas mixture of N_2_ diluted in He in the ChemiSorb 2720 equipment (Micromeritics, Norcross, GA, USA). The SSA was calculated by the single-point method using the Brunauer–Emmet–Teller (BET) equation with the Chemisoft TPx system (version 1.03; data analysis software; Micromeritics, Norcross, GA, USA, 2011). Micrographs and energy-dispersive X-ray (EDX) spectra of the samples were obtained using a JEOL JSM 6400 scanning electron microscope (SEM-EDX) (JEOL, Peabody, MA, USA). The photoactivity of the samples was assessed at λ = 310 nm using the IPW-UV-610 Stainless Steel Inner Sterilizer Light (IPW Industries Inc., Santa Ana, CA, USA), and the amount of cyanide remaining in the solutions was measured at λ = 490 nm with a Jenway 7350 spectrophotometer (Cole-Parmer, Staffordshire, UK).

On the other hand, the point of zero charge (PZC) was determined for all samples under room temperature conditions (19 ± 2 °C) using the pH drift method, where ΔpH = pH_f_ − pH_i_ = 0. The study was performed in 50 mL tubes containing 25 mL of a 0.1 M NaCl solution and 0.1 g of solid sample. The pH levels of the solutions in the tubes were adjusted to values ranging from 3 to 11, using 0.1 M HCl or NaOH solutions. These initial pH values were identified as pH_i_. The tubes were agitated at 250 rpm for 24 h, and the final pH of the supernatant liquid in each tube was measured and noted as pH_f_. The PZC was then determined by plotting ΔpH (ΔpH = pH_f_ − pH_i_) vs. pH_i_. The process was repeated for all nanoparticle samples, using 0.01 and 0.05 M NaCl solutions. The experiments were conducted thrice, and for each sample, the average pH_PZC_ value was informed [102].

### 4.7. Adsorption Studies

In this study, adsorption experiments of cyanide species from aqueous KCN solutions were designed in order to evaluate the effect of pH on adsorption, maximum adsorption capacity, and adsorption thermodynamic and kinetic behaviors. The data obtained from these experiments were fitted to isothermal and kinetic models using the least squares nonlinear regression method [103]. The experiments were performed using the methodology described in previous studies [87,93]. The batch-type reaction system was kept at room temperature, and the pH of the solutions was adjusted to 7.0 ± 0.1 by adding 0.1 M solutions of hydrochloric acid (HCl) or sodium hydroxide (NaOH). The amount of adsorbent material used in all experiments was 200 mg L^−1^. The maximum cyanide adsorption capacity was investigated by varying the concentration of 500 mL of KCN solution from 5 to 200 mg L^−1^. The effect of pH on cyanide adsorption and the thermodynamic and kinetic behaviors of cyanide adsorption were investigated using 500 mL of water containing 20 mg L^−1^ KCN [93]. The alkaline picrate analytical method was used to quantify total cyanide in aqueous solutions [104,105,106]. The remaining cyanide concentration in the solution was determined by UV-vis spectrophotometry at 490 nm, based on the previously prepared calibration curve (R^2^ = 0.9997) according to the Lambert–Beer law. The tests were performed in triplicate, and the results were expressed as the average of three replicates [93]. The procedure was repeated using a cyanide reference solution without extrudates to eliminate any photolysis effects due to natural light. The equation below was utilized to estimate the quantity of cyanide (q_e_) that was adsorbed onto the composites [39]:

(3) $$q_{e}=\left(C_{0}-{\;C}_{e}\right)\times \frac{v}{w}$$

where C_0_ and C_e_ are measured in units of mg L^−1^ and represent the initial and equilibrium concentrations of cyanide, respectively. The mass of the adsorbent (w) is measured in grams (g), and the volume of the solution (v) is measured in liters (L).

The Langmuir, Freundlich, and Temkin isotherm models were utilized to evaluate the total cyanide adsorption at equilibrium. The Langmuir isotherm model can be expressed using the following equation [75]:

(4) $$\frac{C_{e}}{q_{e}}=\frac{1}{K_{L}q_{\max}}+\frac{C_{e}}{q_{\max}}$$

where q_max_ represents the maximum monolayer adsorption and is measured in units of mg g^−1^. K_L_ is the Langmuir constant, which is measured in units of L mg^−1^ and is related to the adsorption energy at equilibrium. C_e_ represents the concentration of solute at equilibrium and is measured in units of mg L^−1^. Moreover, the R_L_ separation factor values can be calculated using the following equation to provide information about the adsorption characteristics [75]:

(5) $$R_{L}=\frac{1}{\left(1+K_{L}C_{e}\right)}$$

The suitability of the adsorption process can be determined based on the R_L_ value as follows: if 0 < R_L_ < 1, it indicates favorable adsorption, while R_L_ > 1 indicates unfavorable adsorption. If R_L_ = 0, it suggests irreversible adsorption, and if R_L_ = 1, it suggests linear adsorption.

Furthermore, the Freundlich isotherm model can be expressed using the following equation [75]:

(6) qe= KFCe1n

where the constant K_F_, which represents the Freundlich constant, is a measure of the adsorption affinity of adsorbents and is expressed in units of L mg^−1^. Another constant, 1/n, specifies the adsorption intensity. In order for adsorption to be favorable, the value of n should fall within the range of 1 to 10 [73].

Temkin isotherm model was also used to describe the adsorption behavior at equilibrium. This model can be expressed using the following equation [42]:

(7) $$q_{e}=q_{\max\;}\ln\left({\mathrm{aC}}_{e}\right)$$

where q_max_ = RT/∆Q and ∆Q = −∆H are evaluated by the Langmuir model, and q_max_ is measured in units of mg g^−1^. C_e_ is the concentration of solute at equilibrium and is measured in units of mg L^−1^, and k_T_ represents the maximum binding energy and is measured in units of mol^−1^.

To conduct thermodynamic studies, the experimental data were analyzed by fitting them to the parameters of the thermodynamic laws that describe Gibbs free energy (∆G^0^, kJ mol^−1^), enthalpy (∆H^0^, kJ mol^−1^), and entropy (∆S^0^, kJ mol^−1^ K^−1^). These laws are commonly represented by the following equation [107]

(8) $$∆G^{0}=-{\mathrm{RT}\;\ln\;k}_{C}\;$$

The van’t Hoff equation was used to relate the thermodynamic parameters ∆G^0^, ∆H^0^, and ∆S^0^, as follows [107]:

(9) $$\ln k_{C}=\frac{-∆H^{0}}{R}\times \frac{1}{T}+\frac{∆S^{0}}{R}$$

where R is the universal gas constant (8.314 J mol^−1^ K^−1^), and T is the absolute temperature (K). The k_C_ is achieved as a dimensionless parameter by multiplying the Langmuir constant (k_L_, L mg^−1^) by a molecular weight of an adsorbate (M_w_, g mol^−1^) and then by factors 1000 and 55.5, which is the number of moles of pure water confined in a liter, as follows: [108]

(10) $$k_{C}={\;k}_{L}\times M_{w}\times 1000\times 55.5\;$$

The analysis of absorption kinetics was carried out using various models, including the pseudo-first-order and pseudo-second-order models, as well as models for intraparticle diffusion, external-film diffusion, and internal-pore diffusion. The pseudo-first-order kinetic model can be expressed by the following equation: [73]:

(11) $$\ln\left(q_{e}-q_{t}\right)=\ln\left(q_{e}\right)-{\;k}_{1}t\;$$

where k_1_ represents the pseudo-first-order rate constant (min^−1^) and the terms q_e_ and q_t_ refer to the amount of cyanide adsorbed per unit weight (mg g^−1^) at equilibrium and at any time t, respectively.

On the other hand, the pseudo-second-order kinetic model can be expressed by the following equation [73]:

(12) $$\frac{t}{q_{t}}=\frac{1}{k_{2}q_{e}^{2}}+\frac{1}{q_{e}}t$$

where k_2_ represents the pseudo-second-order rate constant (g mg^−1^ min^−1^).

Finally, in order to gain a thorough comprehension of how cyanide is adsorbed on the surface of nanoparticles, it is necessary to determine which step in the adsorption process is the rate-limiting one. To do this, the intraparticle diffusion model was utilized. This model assumes that in uniformly mixed solutions, intraparticle diffusion is typically the step that controls the rate of the process. The equation that represents the intraparticle diffusion model is as follows: [73]:

(13) qt= k3t12+A 

where the parameter k_3_ (mg g^−1^ min^−1/2^) represents the rate constant of intraparticle diffusion, while A (mg g^−1^) is a constant indicating the thickness of the boundary layer. A higher value of A indicates a stronger boundary layer effect. When the plot of q_t_ against the square root of time exhibits multiple linear segments, this suggests that the diffusion takes place through multiple stages throughout the process.

The internal-pore diffusion model was also considered in this study to elucidate the kinetic adsorption data. When the adsorption rate is governed by particle diffusion, it is expressed by the following equation [73]:

(14) $$-\ln\left(1-{\left(\frac{q_{t}}{q_{e}}\right)}^{2}\right)=\frac{2\pi^{2}D_{p}}{r^{2}}\;t$$

However, if the adsorption rate is governed by external-film diffusion, it is expressed by the following equation [73]:

(15) $$-\ln\left(1-\left(\frac{q_{t}}{q_{e}}\right)\right)=\frac{D_{f}C_{s}}{{h\;r\;C}_{z}}\;t$$

where q_e_ and q_t_ are expressed in mg g^−1^ and represent the solute amount that the adsorbent phase can take up at the equilibrium and at a particular time, t, respectively. The concentration of ions in the solution and adsorbent phases are represented by C_s_ (mg L^−1^) and C_z_ (mg kg^−1^), respectively, and t (time) denotes the contact time. The average radius of the adsorbent particles is given by r (1 × 10^−7^ m), while h represents the thickness of the film surrounding the particles assumed to be 10^−6^ m in poorly stirred solutions. Furthermore, D_p_ (m^2^ min^−1^) is used to describe the diffusion coefficient in the adsorbent phase, and D_f_ (m^2^ min^−1^) refers to the diffusion in the film phase around the adsorbent particles.

### 4.8. Photodegradation Studies

The procedure described in our previous study [109] was employed to conduct heterogeneous photocatalysis experiments. These experiments were conducted using a box-shaped reactor containing a cylindrical quartz vessel with dimensions of 45 cm in height and 5 cm in diameter, placed in the center of the reactor. Two UVB lamps with a wavelength of 310 nm and power of 15 W were situated on either side of the quartz reaction cylinder, with a distance of 7.5 cm between them. The quartz cylinder had a net volume of 750 mL, and the temperature of the samples within the cylinder was regulated using an air cooling system. The batch method was utilized while maintaining the pH of the solution at 7.0 ± 0.1 through the addition of 0.1 M hydrochloric acid or sodium hydroxide solutions. Typically, 200 mg L^−1^ of composites were magnetically stirred in 500 mL of water containing 20 mg L^−1^ of KCN [93]. All cyanide species photodegradation tests were performed in triplicate. As in the adsorption experiments, the procedure for photodegradation was repeated using a cyanide reference solution without extrudates to eliminate any photolysis effects due to natural light. The photodegradation rate of cyanide species for all composites was monitored using the Langmuir–Hinshelwood equation [110], which can be expressed as follows [46]:

(16) $$\ln\frac{C_{o}}{C_{t}}=k\;K\;t={\;k}_{\mathrm{app}}t$$

where k (min^−1^) and K represent the actual rate constant, and the adsorption constant of the substrate on the composites, respectively. The initial concentration of the substrate is denoted by C_0_ (mg L^−1^), while the concentration at a specific time t (min) is represented by C_t_ (mg L^−1^). The apparent rate constant, k_app_ (min^−1^), can be obtained by plotting ln(C_0_/C_t_) against time t. The slope of the curve-fit line gives the value of the k_app_, and the intercept is zero. 

### 4.9. Reuse of Nanoparticles 

Finally, to confirm the ability of synthesized composites to be reused for cyanide photodegradation, an experiment was conducted to test their recycling potential. The reuse experiment involved five consecutive treatment cycles, where at the end of each cycle, the suspensions were allowed to settle for at least an hour, and the supernatant liquid was removed. The composites were then washed carefully three times with a saturated sodium chloride solution (NaCl 1.5 mol L^−1^) [74]. During each cycle, a fresh solution of KCN (20 mg L^−1^) was treated using 200 mg L^−1^ of composites. This experimental setup was based on previous research [93].

## 5. Conclusions

By utilizing alkaline fusion and hydrothermal synthesis methods, zeolitic material was produced from Ecuadorian clay. This material consisted of FAU-Y, LTA, and NaP1 zeolites and was combined with the precursor clay and ZnTiO_3_/TiO_2_ hybrid semiconductor to create four extrudates (C, TC, ZC, and TZC) that effectively removed cyanide species from aqueous solutions. The study revealed that various factors, such as the chemical composition and properties of the composites, as well as operating conditions (pH, initial concentration of adsorbate, contact time, temperature, and UV exposure), influenced the cyanide removal capacity of the synthesized composites, as previously reported by other authors [111]. Investigating these parameters provided valuable information to identify the optimal operating conditions for the efficient removal of cyanide species from aqueous solutions at a neutral pH. In general, the experimental isotherms aligned with the Langmuir model, which describes monolayer adsorption on a surface with an infinite number of identical sites. The pseudo-second-order kinetic model correlated with this model, indicating a chemisorption process on the surface of the composites under study. Two phenomena were identified that resulted in the rapid adsorption of cyanide species until surface saturation. The first suggests the exchange of cationic species in the active centers of the extruded composites with the cyanide species, while the second supports the formation of cyanide complexes that increase the adsorption of these species on the surface of the extruded composites. The synergistic coupling of adsorption and photocatalysis processes significantly improved the removal capacity of cyanide species from aqueous solutions of the C, TC, ZC, and TZC composites. In general, the efficiency of cyanide removal depends to a large extent on the nature of the material and the components added to it, such as zeolites and photocatalysts. The optimization of wastewater treatment materials and processes is essential to achieve high efficiency in the removal of cyanide and other contaminants. On the other hand, although materials in the form of extrudates may have a lower cyanide adsorption and photodegradation capacity compared to powdered materials, their advantages in terms of handling, mechanical resistance, durability, and stability in the environment may justify their use. use in certain practical applications. However, the choice of material in extruded or powder form will depend on the specific needs of each application.

Finally, this study confirmed the transformation of a natural resource such as clay into high-value zeolites and their potential for removing cyanide species from aqueous solutions, which could lead to the development of clean technologies on an industrial scale using available natural resources.

## Figures and Tables

**Table 1:** Composition (%) of clay, zeolite, and composites.

Compound	Al_2_O_3_	SiO_2_	K_2_O	CaO	Fe_2_O_3_	TiO_2_	ZnO
Zeolite	19.62 (±0.79)	41.65 (±0.68)	0.78 (±0.02)	1.22 (±0.01)	0.80 (±0.01)	-	-
C	21.10 (±0.85)	45.30 (±0.63)	1.14 (±0.02)	3.11 (±0.02)	2.44 (±0.01)	-	-
ZC	15.70 (±0.79)	41.80 (±0.63)	1.34 (±0.03)	2.96 (±0.02)	2.38 (±0.01)	-	-
TC	11.60 (±1.56)	23.90 (±0.58)	0.38 (±0.03)	1.61 (±0.02)	2.64 (±0.03)	51.40 (±0.14)	20.66 (±0.06)
TZC	12.10 (±1.31)	37.60 (±0.68)	1.58 (±0.04)	2.63 (±0.03)	2.94 (±0.02)	31.30 (±1.82)	16.30 (±0.07)

**Table 2:** Elemental analysis (wt%) for clay, zeolite, ZnTiO_3_/TiO_2_, and TZC composite.

	C	O	Si	Al	Fe	Na	Ca	K	Zn	Ti
Clay	12.12	50.85	29.30	4.24	0.78	1.17	0.92	0.61	-	-
Zeolite	20.77	50.91	14.44	6.77	0.88	3.54	2.05	0.62	-	-
ZnTiO_3_/TiO_2_	5.42	33.60	-	-	-	-	-	-	6.13	54.85
TZC	5.05	41.84	7.59	4.55	0.49	0.84	0.45	0.36	10.19	28.64

**Table 3:** Specific surface area (SSA) (m^2^/g) and point of zero charge (pH_PZC_) of the compounds.

Compound	Composition	SSA (m^2^ g^−1^)	pH_PZC_
Zeolite (P)	Zeolite	386	8.4
ZnTiO_3_/TiO_2_ (P)	ZnTiO_3_/TiO_2_	88	7.0
Clay C (P)	Clay	22	6.8
Composite ZC (P)	Zeolite + Clay	184	7.8
Composite TC (P)	ZnTiO_3_/TiO_2_ + Clay	45	6.9
Composite TZC (P)	ZnTiO_3_/TiO_2_ + Zeolite + Clay	62	7.3
Clay C (E)	Clay	12	6.8
Composite ZC (E)	Zeolite + Clay	125	7.8
Composite TC (E)	ZnTiO_3_/TiO_2_ + Clay	36	6.9
Composite TZC (E)	ZnTiO_3_/TiO_2_ + Zeolite + Clay	48	7.3

**Table 4:** Isotherm parameters for cyanide adsorption on extruded compounds at different temperatures.

Isotherm Parameters		293.15 K				298.15 K				303.15 K			
		C	TC	ZC	TZC	C	TC	ZC	TZC	C	TC	ZC	TZC
Langmuir	q_max_	56.92	67.73	73.37	71.83	62.61	74.50	80.71	79.01	68.30	81.28	88.04	83.20
	(mg g^−1^)	(±1.41)	(±1.36)	(±1.53)	(±1.74)	(1.52)	(2.11)	(3.52)	(3.10)	(1.52)	(2.11)	(3.52)	(3.10)
	K_L_	0.14	0.16	0.18	0.23	0.13	0.15	0.38	0.33	0.28	0.35	0.46	0.41
	(L mg^−1^)	(±0.03)	(±0.02)	(±0.04)	(±0.03)	(0.03)	(0.04)	(0.05)	(0.04)	(0.03)	(0.04)	(0.05)	(0.05)
	R_L_	0.57	0.52	0.37	0.49	0.18	0.15	0.12	0.13	0.15	0.13	0.10	0.11
	χ^2^	1.67	1.90	1.80	1.41	2.45	3.97	2.21	2.89	2.51	3.97	2.912	2.89
	R^2^	0.96	0.98	0.99	0.98	0.99	0.98	0.97	0.95	0.99	0.98	0.97	0.95
Freundlich	K_F_	6.25	8.57	2.56	15.26	6.88	9.43	2.82	16.79	7.50	10.28	3.07	18.31
	(L mg^−1^)	(±1.20)	(±1.38)	(±2.05)	(±1.69)	(1.94)	(2.25)	(2.59)	(2.24)	(1.84)	(2.25)	(2.04)	(2.24)
	n	2.35	2.44	2.54	2.52	2.58	2.68	2.79	2.77	2.82	2.93	2.59	3.02
		(±0.43)	(±0.40)	(±0.43)	(±0.30)	(0.39)	(0.41)	(0.44)	(0.44)	(0.39)	(0.41)	(0.35)	(0.44)
	1/n	0.42	0.41	0.39	0.40	0.41	0.37	0.36	0.36	0.39	0.34	0.44	0.33
	χ^2^	3.34	5.47	3.67	5.18	4.70	3.96	4.15	3.95	4.97	3.96	4.15	3.95
	R^2^	0.87	0.90	0.94	0.90	0.92	0.89	0.86	0.84	0.92	0.89	0.86	0.84
Temkin	q_max_	12.53	14.10	19.80	14.12	14.03	16.21	21.98	15.96	16.97	19.74	26.70	17.47
	(mg g^−1^)	(±0.96)	(±0.94)	(±0.19)	(±1.12)	(±1.06)	(±0.99)	(±1.19)	(±1.02)	(±1.11)	(±1.21)	(±1.23)	(±1.16)
	a	0.36	0.52	0.71	0.53	0.45	0.60	0.87	0.65	0.51	0.67	0.92	0.69
	(mol^−1^)	(±0.07)	(±0.09)	(±0.06)	(±0.16)	(±0.05)	(±0.05)	(±0.07)	(±0.09)	(±0.07)	(±0.09)	(±0.06)	(±0.10)
	χ^2^	13.9	16.47	16.75	12.72	11.7	12.31	13.14	10.05	10.8	11.23	15.13	12.15
	R^2^	0.95	0.97	0.90	0.95	0.96	0.95	0.92	0.91	0.97	0.96	0.95	0.90

**Table 5:** Thermodynamic parameters of the cyanide adsorption on extruded compounds.

Samples	Temperature (K)	ln k_C_	∆G° (kJ mol^−1^)	∆H° (kJ mol^−1^)	∆S° (kJ mol^−1^ K^−1^)
C	293.15	12.56	−30.61	−29.93	0.21
	298.15	12.73	−31.56		
	303.15	12.96	−32.67		
TC	293.15	12.81	−31.22	−26.40	0.20
	298.15	12.96	−32.12		
	303.15	13.17	−33.19		
ZC	293.15	13.11	−31.95	−24.83	0.19
	298.15	13.25	−32.84		
	303.15	13.45	−33.89		
TZC	293.15	12.96	−31.59	−27.42	0.20
	298.15	13.11	−32.50		
	303.15	13.33	−33.61		

**Table 6:** Kinetic parameters for cyanide adsorption on extruded compounds.

Kinetic Parameters		C	TC	ZC	TZC
Pseudo-first-order	q_max_ (mg g^−1^)	11.47 (±2.17)	13.73 (±1.81)	17.98 (±2.21)	14.93 (±2.26)
	k_1_ (L mg^−1^)	0.02 (±6.01 × 10^−4^)	0.03 (±4.93 × 10^−4^)	0.03 (±4.85 × 10^−4^)	0.02 (±1.02 × 10^−3^)
	χ^2^	2.11	2.38	2.69	2.13
	R^2^	0.99	0.99	0.99	0.98
Pseudo-second-order	q_max_ (mg g^−1^)	13.63 (±1.15)	16.42 (±1.24)	21.55 (±1.67)	17.10 (±2.00)
	k_2_ (L mg^−1^)	1.06 × 10^−3^ (±5.24 × 10^−5^)	8.32 × 10^−4^ (±5.16 × 10^−5^)	6.41 × 10^−4^ (±8.50 × 10^−5^)	1.22 × 10^−3^ (±6.92 × 10^−5^)
	χ^2^	2.13	2.74	3.07	2.56
	R^2^	1.00	1.00	1.00	1.00
Intraparticle diffusion	k_3_ (mg g^−1^ min^−1/2^)	0.53 (±0.01)	0.64 (±0.02)	0.84 (±0.02)	0.65 (±0.01)
	*A*	1.65 (±0.17)	1.82 (±0.22)	2.56 (±1.01)	3.33 (±1.15)
	R^2^	0.92	0.91	0.89	0.87
External-film diffusion	Df (m^2^ min^−1^)	5.97 × 10^−13^	7.43 × 10^−13^	1.15 × 10^−12^	1.02 × 10^−12^
	R^2^	0.99	0.98	0.98	0.99
Internal-pore diffusion	Dp (m^2^ min^−1^)	4.00 × 10^−18^	4.20 × 10^−18^	5.30 × 10^−18^	5.70 × 10^−18^
	R^2^	0.98	0.96	0.95	0.97

**Table 7:** Comparison of the adsorption capacity (mg g^−1^) of various materials for cyanide removal.

Adsorbent	q_max_ (mg g^−1^)	Isotherm Model	Kinetic Model	Reference
ZnTiO_3_	57.32	Langmuir	Pseudo-second-order	[93]
La/ZnTiO_3_	59.22	Langmuir	Pseudo-second-order	[93]
Ce/ZnTiO_3_	42.00	Langmuir	Pseudo-second-order	[93]
TiO_2_	46.48	Langmuir	Pseudo-second-order	[87]
La/TiO_2_	54.96	Langmuir	Pseudo-second-order	[87]
Ce/TiO_2_	51.39	Langmuir	Pseudo-second-order	[87]
Eu/TiO_2_	49.25	Langmuir	Pseudo-second-order	[87]
ZnO	275.00	Langmuir	Pseudo-second-order	[75]
NiO	185.00	Langmuir	Pseudo-first-order	[75]
ZnO-NiO	320.00	Langmuir	Pseudo-second-order	[75]
LTA zeolite modified with HDMTMAB	24.09	Langmuir	-	[73]
Clay-K	253.98	-	Pseudo-second-order	[72]
TiO_2_/Fe_2_O_3_	124.87	-	Pseudo-second-order	[72]
Fe-MFI zeolite	33.98	Langmuir	Pseudo-second-order	[94]
C	56.92	Langmuir	Pseudo-second-order	This study
TC	67.73	Langmuir	Pseudo-second-order	This study
ZC	73.37	Langmuir	Pseudo-second-order	This study
TZC	71.83	Langmuir	Pseudo-second-order	This study

**Table 8:** Comparison of the photodegradation efficiency (%) of various materials for cyanide removal.

Material	[CN] (mg L^−1^)	[Catalyst] (g L^−1^)	Time (min)	Efficiency (%)	Reference
ZnTiO_3_	20.0	0.2	90	90.7	[93]
La/ZnTiO_3_	20.0	0.2	90	98.5	[93]
Ce/ZnTiO_3_	20.0	0.2	90	95.1	[93]
TiO_2_	20	0.2	90	84	[87]
La/TiO_2_	20	0.2	90	97	[87]
Ce/TiO_2_	20	0.2	90	89	[87]
Eu/TiO_2_	20	0.2	90	86	[87]
TiO_2_/Fe_2_O_3_/zeolite	200	1.4	160	89	[46]
TiO_2_/Fe_2_O_3_/PAC	300	1.4	170	97	[46]
Blast furnace sludge (BFS)	750	2.0	120	97	[72]
Cts-Ag	71.6	2.5	180	98	[95]
Fe^2+^	10	0.14	30	86	[96]
TiO_2_	30	0.05	60	72	[97]
Co/TiO_2_/SiO_2_	100	2.0	60	55	[98]
TiO_2_/SiO_2_	100	1.7	180	93	[99]
Ce/ZnO	250	4.0	180	84	[100]
C	20	0.2	60	48	This study
TC	20	0.2	60	91	This study
ZC	20	0.2	60	48	This study
TZC	20	0.2	60	86	This study

## Data Availability

Data are contained within the article.
